# Halite crystallization at Sfax solar salterns, Tunisia, driven by brine geochemistry and biogeochemical interactions

**DOI:** 10.3389/fmicb.2026.1843317

**Published:** 2026-10-13

**Authors:** Najoua Gharsalli, Najla Mhiri, Chokri Yaich, Fatma Karray

**Affiliations:** 1 Laboratory of Environmental Bioprocesses, Centre of Biotechnology of Sfax, Sfax, Tunisia; 2 Laboratory of Environmental Engineering and Ecotechnology (GEET), University of Sfax, Sfax, Tunisia; 3 Laboratory of Georesources, Materials, Environment and Global Change (GEOGLOB), Sfax, Tunisia; 4 National Engineering School of Sfax (ENIS), University of Sfax, Sfax, Tunisia

**Keywords:** archaea, biogeochemical interactions, biomineralization, brine geochemistry, halite crystallization, hypersaline environments

## Abstract

Solar salterns are hypersaline systems characterized by evaporation-driven salinity gradients that promote the precipitation of evaporite minerals. Here, we investigate the influence of halophilic microbial communities on halite crystallization along the salinity gradient of the Sfax solar saltern (Tunisia). Geochemical analyses of 26 brine samples (mean salinity range ~28–570 g L^−1^) demonstrated marine-fed evaporative sequences, allowing the selection of three representative basins along a gradient of 150 g/L to 280 g/L (M22, B22, and TS5 sites). Enrichment cultures inoculated with surface (SB) and bottom (BB) brines were established under conditions reproducing *in situ* brine chemistry. Three halophilic archaeal species, *Halorubrum xinjiangense, H. africanae*, and *H. ezzemoulense*, were isolated from cultures able to tolerate salinities of up to 280 g L^−1^. Subsequently, controlled crystallization experiments using high-salinity biomass as a progressively increasing inoculum revealed that biomass-associated microbial populations influenced halite precipitation kinetics, yield, and crystal morphology relative to abiotic controls. SB-derived cultures reached maximum halite-dominated precipitate mas at a lower biomass level (25% inoculum), yielding 5.550 ± 0.127 g after 10 days at 0.555 ± 0.013 g d^−1^, whereas BB-derived cultures peaked at 75% inoculum (64.20 ± 2.632 mg mL^−1^), producing 3.570 ± 0.085 g after 12 days at 0.299 ± 0.028 g d^−1^. This contrast shows that biomass affects crystallization differently in surface- and bottom-derived cultures, with differences in crystal size, shape, aggregation, and uniformity. These results demonstrate that halophilic microorganisms could modulate halite crystallization under extreme salinity, highlighting the importance of microbial processes in evaporite mineralization within hypersaline environments.

## 1 Introduction

Evaporites are a class of sedimentary rocks that originate primarily from the evaporation of hypersaline waters in both marine and lacustrine settings. The oldest known evaporite formations on Earth date back to the late Archean to early Proterozoic eras, approximately 2.5 to 2.0 billion years ago (Moore, 1960; González-Esvertit et al., 2023). They occupy a geologically significant interface between the hydrosphere and the Earth's silicate crust. Mineralogically, they are composed of soluble ionic compounds such as K^+^, Na^+^, Ca^2+^, Mg^2+^, Cl^−^, ${\text{SO}}_{4}^{2-}$, and ${\text{CO}}_{3}^{2-}$ (Warren, 2016). Because relatively weak electrostatic interactions bind these ions, evaporite minerals are highly susceptible to dissolution and diagenetic alteration. Owing to these characteristics and their depositional contexts, evaporites are recognized as valuable archives for reconstructing paleoenvironmental and hydrological conditions.

Building upon this geological perspective, hypersaline ecosystems where evaporite formation frequently occurs are not only of geochemical interest but also represent unique ecological niches at the limits of habitability. These environments encompass salt flats, chotts, solar salterns, coastal marshes, lagoons, and inland saline ponds. They are geographically widespread, occurring for example, across the Mediterranean basin in Tunisia (Guermazi et al., 2024; Mahfoudhi et al., 2025; Lakhdar and Talbi, 2025; Boukhalfa et al., 2025), Algeria (Chamekh et al., 2019; Aoudia et al., 2020; Hafhouf et al., 2022 Amiour et al., 2022); Spain (Bourhane et al., 2023; Fairén et al., 2023; García-Roldán et al., 2023), North America (Keevil et al., 2022; Daoust et al., 2023; Robertson et al., 2025), Arabian Gulf (Malah et al., 2023; Mor et al., 2024; He et al., 2024) and sub-Saharan Africa (Angulo et al., 2025; Maghembe et al., 2025). The salinity of these systems far exceeds that of seawater (3.5% w/v) (Oren, 1999), thereby imposing extreme osmotic stress.

Despite such harsh conditions, hypersaline habitats host diverse halophilic microorganisms, considered among the most ancient and resilient forms of life on Earth. These extremophiles have evolved sophisticated adaptations, including the synthesis of compatible solutes to stabilize intracellular structures and the production of acidic proteins that retain enzymatic function under high salinity. Although elevated temperature and nutrient availability can enhance microbial activity, abiotic constraints such as salinity fluctuations, high ultraviolet radiation, and alkaline pH severely restrict biodiversity, favoring only a limited but robust spectrum of taxa (Oren, 2002; Jaakkola et al., 2016).

Beyond their ecological significance, hypersaline environments constitute powerful natural laboratories for geobiological research. They provide opportunities to explore microbial evolution, adaptation, and biomineralization processes. Halophilic microorganisms are also an important source for industrial and environmental applications. They produce a wide range of salt-tolerant enzymes such as amylases, proteases, cellulases, and lipases that remain active under high salinity and alkaline conditions, making them attractive for use in food processing, pharmaceuticals, textiles, biofuels, and wastewater treatment (Setati, 2010; Kumar et al., 2012; Yin J. et al., 2015; Yin S. et al., 2015; Edbeib et al., 2016; Baati et al., 2022; Ben Hamad Bouhamed et al., 2024; Essoufi et al., 2024). Furthermore, they synthesize commercially valuable metabolites, including ectoine, widely used in cosmetics and medicine (Lentzen and Schwarz, 2006), and bacteriorhodopsin, a protein with applications in bioelectronics and solar energy conversion (Seyedkarimi et al., 2015; Kanekar et al., 2021; Steffy et al., 2022). The discovery of antimicrobial peptides such as halocins further underscores their biotechnological promise (Rodríguez-Valera et al., 1982; Delgado-García et al., 2014).

At the same time, hypersaline environments hold considerable astrobiological relevance, as they serve as terrestrial analogs for extreme settings on other planetary bodies. The coexistence of extremophiles with distinctive geochemical processes offers critical insights into biosignature preservation and aqueous alteration. Studies of sebkhas (Diloreto et al., 2023), coastal lagoons (Boidi et al., 2022; Fairén et al., 2023), deep-sea hypersaline anoxic basins (Fisher et al., 2021), and Antarctic brines (Buffo et al., 2022; Guglielmin et al., 2023) contribute to understanding the potential for extraterrestrial life in similar hypersaline systems.

Evaporitic minerals such as gypsum and carbonates are central to this discussion. Gypsum, for instance, is particularly effective at preserving organic matter and biosignatures, making it a strong analog for Martian and icy moon environments. Deposits from hyperarid sites like Qatar, the Atacama Desert, and Sorbas have provided models for microbial survival under conditions of desiccation and intense UV stress (Cai et al., 2012; Saini et al., 2023). The discovery of gypsum at Martian sites such as Meridiani Planum and Gale Crater has reinforced hypotheses regarding evaporitic processes and transient habitability on Mars (Clark et al., 2005; Rapin et al., 2019). Current rover missions, such as NASA's Perseverance equipped with SHERLOC and SuperCam, further enhance the capacity to detect organics and mineralogical biosignatures in Martian evaporites (Bhartia et al., 2021; Farley et al., 2022). Similarly, carbonate precipitation in hydrothermal settings like terrestrial hot springs offers analogs for potential subsurface life-supporting systems on Mars and icy ocean worlds such as Europa and Enceladus, where hydrothermal activity may sustain microbial communities (Fox-Powell et al., 2016; Pontefract et al., 2017).

Within this broader framework, hypersaline systems can be divided into two categories: natural environments, including sebkhas, saline lakes, and lagoons, and artificial systems, such as solar salterns constructed for industrial salt production. These thalassohaline environments are distributed globally along tropical and subtropical coasts in arid and semiarid regions (Gammoudi et al., 2022; Bonifazi et al., 2023; Eckmann et al., 2023 Liébana et al., 2024; Kim and Park, 2024). Unlike natural habitats, salterns provide controlled yet dynamic physicochemical gradients that simulate many of the processes occurring in natural hypersaline systems.

In the Mediterranean basin, solar salterns are widely distributed (Gammoudi et al., 2022; Bonifazi et al., 2023; Altahan and Nower, 2023; Rodríguez-Valdecantos et al., 2023; Liébana et al., 2024), particularly in regions with arid to semi-arid climates. These conditions enable extended periods of salt production through solar evaporation. Besides their economic role, salterns constitute valuable ecological models for studying microbial diversity and adaptation strategies in hypersaline ecosystems. Importantly, the pond networks of salterns exhibit strong salinity gradients, creating a series of interconnected ecological niches. Each niche selects microbial taxa with distinct salt tolerances, thereby providing unique opportunities to investigate the adaptive mechanisms of halophiles along salinity gradients (Konstantinidis et al., 2022).

Against this background, the Sfax solar salterns in Tunisia represent an especially compelling case study. These environments are shaped by pronounced physicochemical stratification, making them ideal for examining the interplay between physical, chemical (Amdouni, 2006, 2009), and biological processes (Masmoudi et al., 2019; Gammoudi et al., 2022; Guermazi et al., 2024). Their steep salinity gradients, high evaporation rates, and microbial diversity render them open-air laboratories for studying microbially mediated salt crystallization and the geobiological drivers of evaporite formation.

The Sfax solar salterns also hold ecological importance, as they support rich biodiversity. More than 100 bird species, including *Tringa totanus, Larus genei*, and *Sternula albifrons*, are found in these habitats (Chokri and Selmi, 2011). Aquatic communities are equally diverse, encompassing marine algae (Gammoudi et al., 2022), phytoplankton (Ayadi et al., 2002, 2004), benthic invertebrates (Guermazi et al., 2024), and the brine shrimp *Artemia salina*, which plays a pivotal role in hypersaline food webs (Toumi et al., 2005; Aloui et al., 2012). Along the salinity gradient, microbial communities shift from cyanobacteria and sulfur bacteria in low-salinity zones to halophilic archaea in extreme salinity ponds, contributing to nutrient cycling and sustaining *Artemia* populations. High-salinity ponds are further dominated by *Dunaliella viridis* and *D. salina*, whose carotenoid production, together with bacterioruberin pigments from halobacteria, drives the striking coloration of the brines (Ayadi et al., 2002, 2004).

However, despite this well-established ecological framework, the mechanisms controlling halite crystallization, especially the interaction between geochemical evolution and microbial activity, are still insufficiently studied in the Sfax solar salterns and are largely overlooked in similar Mediterranean and North African saltworks. This highlights the originality and the importance of this research. The study adopts an interdisciplinary approach integrating geochemistry and geomicrobiology to investigate the biogeochemical dynamics of the Sfax solar salterns. Specifically, it aims to (i) characterize the geochemical properties of the brines based on the analysis of 26 samples collected across the salterns; (ii) isolate and identify halophilic microorganisms inhabiting these hypersaline environments through enrichment cultures established from three representative sites spanning moderate, high, and extreme salinity conditions; and (iii) investigate the effects of extreme-halophile consortia on halite formation and biomineralization under controlled crystallization conditions.

## 2 Study area

### 2.1 Geographical features

The Sfax solar salterns, also known as the Thyna salt pans, constitute an important ecological and industrial system located near Sfax on the southeastern coast of Tunisia (Figures 1A, B). Spanning roughly 1,500 hectares, the site comprises a network of interconnected evaporation basins engineered for the progressive concentration and crystallization of seawater. Situated along the Ras Sfax peninsula, the marshes stretch approximately 12 kilometers parallel to the Mediterranean Sea and are shielded from direct marine influence by a 4-meter-high embankment made of compacted red silt (Figures 1B, C). Basin depths range from 20 to 70 cm, generating hypersaline environments with salinity levels between 40 and 400 gL^−1^. These conditions not only facilitate substantial salt production but also provide a vital habitat for a diverse array of halophilic microorganisms and local wildlife.

**Figure 1:**
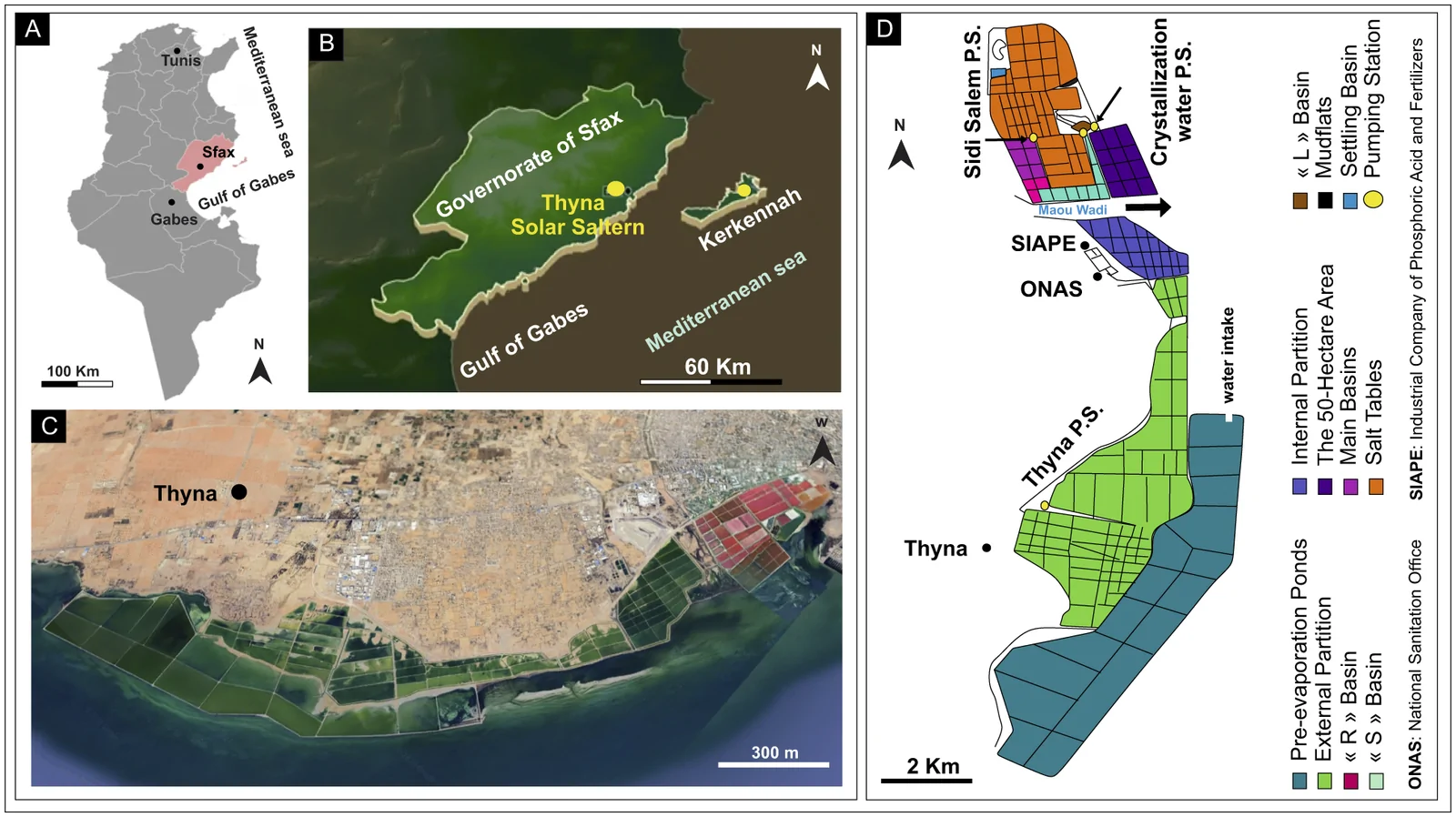
Location of the study area. **(A)** Geographical position of Sfax within the Mediterranean Basin; **(B)** Location of the solar salterns in the Sfax Governorate; **(C)** Satellite view of the Sfax solar salterns; and **(D)** Schematic diagram showing the different evaporation basins of the saltworks.

### 2.2 Environmental features

Located within the Gulf of Gabès, one of the few Mediterranean regions experiencing tidal variations of up to 2 meters (Figure 1A), the Sfax solar salterns encompass a mosaic of shallow marine waters, intertidal marshes, and anthropogenic salt pans (Figures 1C, D). This distinctive tidal regime promotes the formation of natural salt flats, a habitat that has become increasingly rare in Tunisia. The area is subject to semidiurnal tides, with amplitudes reaching up to 2.3 m during spring tides (Sammari et al., 2006).

### 2.3 Evaporation and crystallization processes in saline ponds

The Sfax solar salterns (Figure 1D), serve as both a vital wetland and a major industrial salt production site, yielding around 300,000 tons of salt annually. Salt formation begins in the pre-ponds, where seawater undergoes controlled evaporation and salinity rises from 41 to 75‰, aided by shallow depths and cyanophyte mats that enhance water loss (Figure 1D). The brine then flows through sequential ponds, gradually reaching 130‰ in the outer partitions, 19 °B in the inner partitions, and finally 300‰ in the crystallization tables, where halite precipitates (Figure 1D). Sedimentary facies evolve progressively with increasing salinity, from detrital and biodetrital facies (< 70‰) to carbonate-rich facies (70‰−170‰), gypsum-carbonate facies (170‰−250‰), and gypsum-halite facies (250‰−280‰). Beyond 300‰, pure halite dominates, while magnesium salts accumulate along pond margins (Figure 1D). Altogether, this system reflects the interplay of biological, chemical, and hydrological processes, ensuring efficient salt extraction and byproduct recovery.

## 3 Materials and tools

### 3.1 Sampling

Twenty-six brine samples (Figure 2) were collected from the Sfax solar salterns (34°39′N, 10°43′E) in May 2019 as part of an integrated geochemical and geomicrobiological survey. Sampling covered a mean salinity gradient ranging from ~28–570 g L^−1^, capturing the transition from seawater-fed concentration ponds to the highly evaporative post-crystallization basins. Surface brines were retrieved using sterile 1-L glass flasks along the main hydrological units of the saltern system. Immediately after collection, samples were stored in insulated containers, transported to the laboratory, and kept at 4 °C pending geochemical measurements and subsequent microbiological analyses. All samples were collected in duplicate to ensure analytical reproducibility.

**Figure 2:**
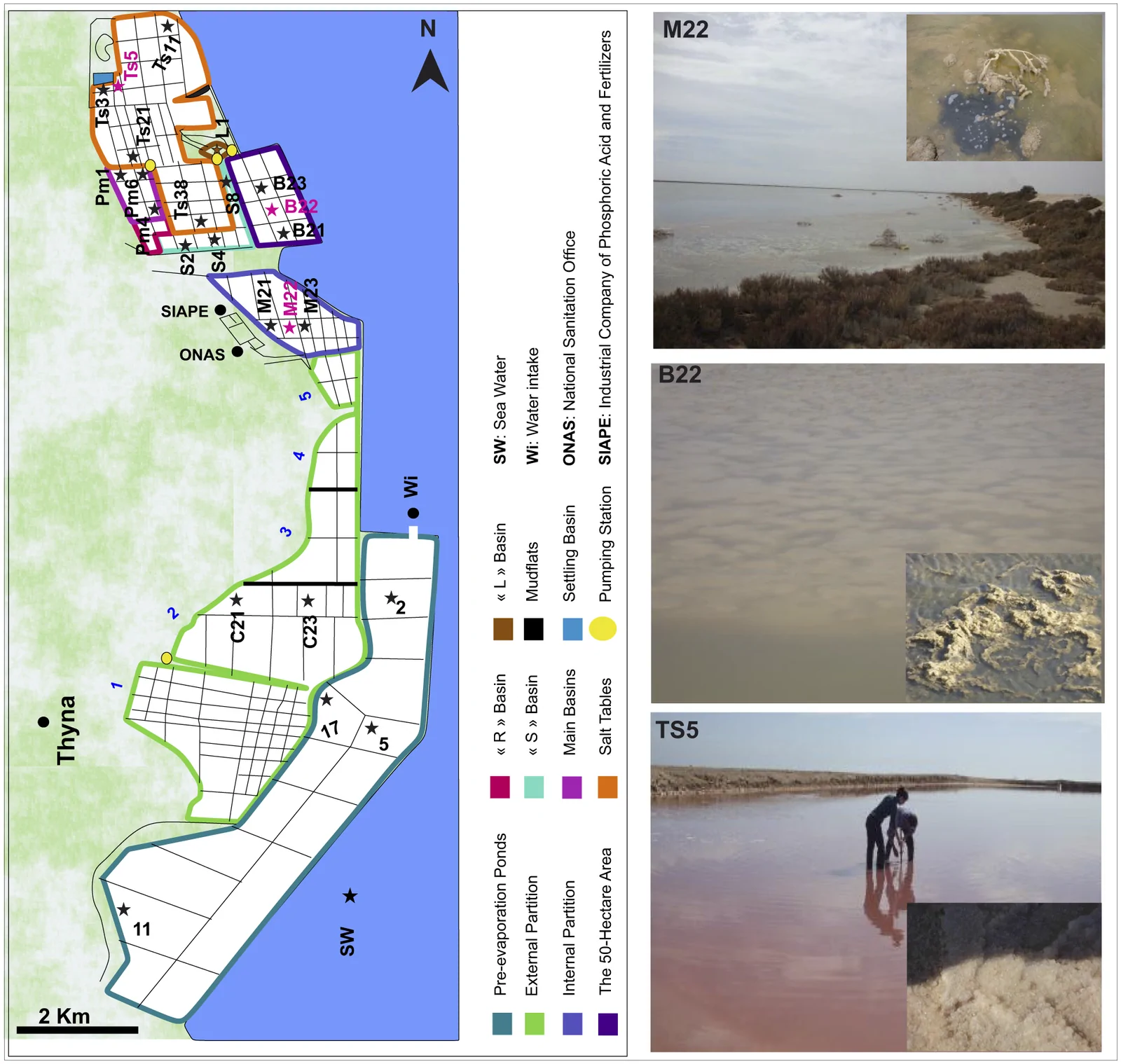
Spatial distribution of sampling sites across the Sfax solar saltern basins. All collected surface brine samples (SW, Wi, 2, 5, 11, 17, C21, C23, M21, M22, M23, B21, B22, B23, Pm1 Pm4, Pm6, TS3, TS5, TS11, TS21, TS38, S2, S4, S8 and L1; see Table 1) underwent comprehensive geochemical analysis. Samples M22, B22, and TS5 (highlighted in pink) were further selected for microbiological investigation due to their representative mineralogical and salinity profiles (see Supplementary Table 1). Two types of samples, SB and BB, were collected from each site, and photographs were taken to document the locations. The corresponding sedimentary facies are as follows: (i) M22—a thin gypsum layer overlying carbonate-rich mud; (ii) B22—a well-developed gypsum bed; and (iii) TS5—a surface halite crust representing the terminal stage of evaporation and salt precipitation. The asterisks (*) indicate the locations of the sampling sites.

For microbiological investigations, six representative brines were selected from three key sites, M22, B22, and TS5 (Figures 2, 3). At each site, surface brine (SB) and bottom brine (BB) were sampled independently, the latter corresponding to the denser layer in direct contact with basin sediments. The overlying water column above BB samples ranged from 14 to 61 cm, depending on basin morphology (Table 1). All microbiological samples were handled aseptically, transported under cold conditions, and stored at 4 °C until processing.

**Figure 3:**
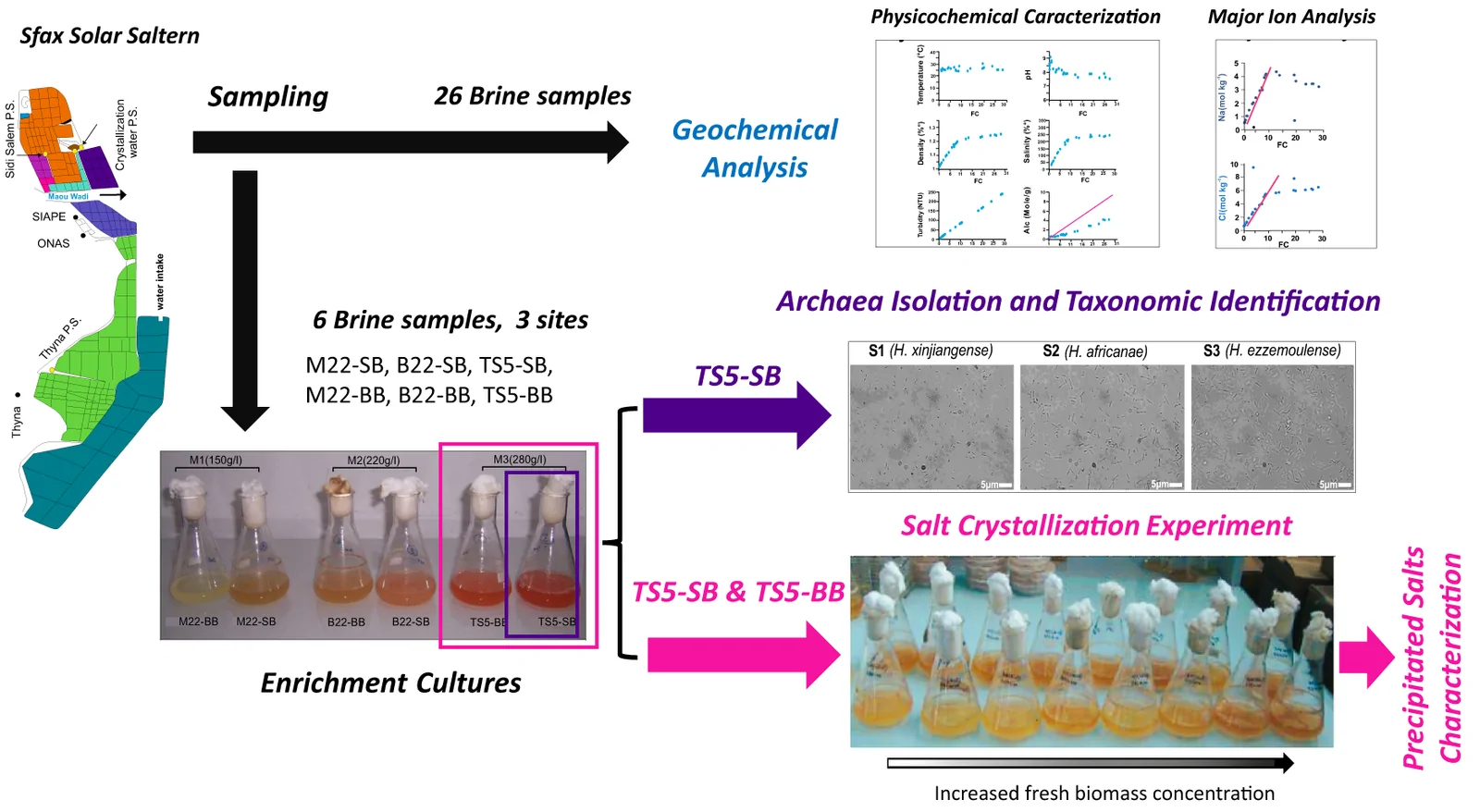
Experimental workflow: hypersaline brine sampling and geochemical characterization, microbial enrichment, archaeal strain isolation and taxonomic identification, salt precipitation experiments, and characterization of the resulting precipitates.

**Table 1:** Physicochemical characteristics of three representative brine samples: moderate salinity (M22), high salinity (B22), and hypersaline conditions (TS5) used for microbial studies and halite precipitation experiments.

Parameter	M22	B22	TS5
Major elements/ions			
${\text{CO}}_{3}^{2-}$ (g L^−1^)	0.003	0.002	0.000
${\text{HCO}}_{3}^{-}$ (g L^−1^)	0.037	0.049	0.220
Ca (g L^−1^)	1.213	0.638	0.234
${\text{SO}}_{4}^{2-}$ (g L^−1^)	14.358	17.650	54.307
Na (g L^−1^)	51.667	68.325	70.000
Cl (g L^−1^)	107.183	133.592	191.690
Mg (g L^−1^)	9.478	10.052	37.242
K (g L^−1^)	2.267	3.267	10.867
Physicochemical parameters			
Density (g mL^−1^)	1.123	1.176	1.247
pH	8.28	7.89	7.65
T (°C)	27	24	27
TSS (g L^−1^)	0.1	0.1	0.9
Layer thickness (cm)	37	61	14
Derived parameters			
Salinity (g L^−1^)	186.206	233.575	364.560
NaCl (g L^−1^)	158.850	201.917	261.690
Environmental setting			
Basin type	Internal partition	The 50-hectare area	Salt tables
Facies	Gypsum-carbonate	Gypsum	Halitic

### 3.2 Geochemical analysis

A total of 26 brine samples were subjected to both field and laboratory analyses. Field measurements included water depth and temperature, while laboratory determinations focused on density, salinity, pH, turbidity, and major ion composition.

#### 3.2.1 Physicochemical parameters

Field temperatures were recorded using a BRAVO mercury thermometer. In the laboratory, brine densities were measured with a hydrometer in a graduated cylinder, and samples were filtered through 0.45 μm membrane filters prior to analysis. pH was measured using a METROHM bench meter calibrated with standard buffer solutions (pH 7.0 and 4.0; Figure 3).

#### 3.2.2 Major elements

Major ions and alkalinity were determined using standard analytical procedures (Figure 3). Calcium, magnesium, sodium, and potassium concentrations were measured by atomic absorption spectrometry (AAS). Chloride was analyzed by argentometric titration following the Mohr method, using standardized AgNO_3_ and potassium chromate as the endpoint indicator, while sulfate was quantified gravimetrically as barium sulfate (BaSO_4_). Alkalinity analyses were performed within 24 h of sampling. Samples containing residual free chlorine were pre-treated with 0.1 N sodium thiosulfate. Total alkalinity was determined by titration with standardized HCl using phenolphthalein, and total alkalinity capacity was subsequently measured using methyl orange as the endpoint indicator.

#### 3.2.3 Choice of concentration factor

The evolution of physicochemical parameters (temperature, density, salinity, pH, and alkalinity) and major ion concentrations (Na, Ca, Mg, K, ${\text{SO}}_{4}^{2-}$, and Cl^−^) was evaluated using a concentration factor (FC) as a reference scale. The FC was calculated based on potassium concentrations measured in seawater from the Sfax coast and in brine samples collected from the different ponds of the Sfax solar saltern. Potassium was selected as a conservative tracer due to its low chemical reactivity and its negligible involvement in evaporitic mineral assemblages during evaporation up to salinities of approximately 300‰. The concentration factor was defined as FC = Ci/CEM, where Ci is the potassium concentration in the sample, and CEM is the potassium concentration in seawater.

### 3.3 Biological analysis

#### 3.3.1 Enrichment cultures and isolation of halophilic microorganisms

The enrichment and isolation of aerobic halophilic microorganisms were conducted using three distinct culture media prepared in 250 mL Erlenmeyer flasks, each containing 100 mL of liquid medium adjusted to specific salt concentrations of 150 g/L (M1), 220 g/L (M2), and 280 g/L (M3; Table 2, Figure 3). The M1, M2, and M3 media approximately mimicked the low-, moderate-, and high-NaCl conditions of M22, B22, and TS5, respectively (Table 1, Supplementary Table S1). Table 2 summarizes the complete composition of each medium. All media were prepared and inoculated with 5 mL of surface brine (SB) and 5 mL of bottom brine (BB) collected from M22, B22, and TS5 sites located in the Sfax solar salterns (Table 1, Figure 2, Supplementary Figures S2, S3). Negative control was systematically included to verify sterility and exclude potential background growth. The enrichment cultures M22-SB, M22-BB, B22-SB, B22-BB, TS5-SB, and TS5-BB were then incubated at 37 °C under continuous agitation (160 rpm) for 10 days.

**Table 2:** Chemical composition of the different culture media (M1, M2, and M3) used in this study.

Components	Medium 1 (M1)	Medium 2 (M2)	Medium 3 (M3)
NaCl (g L^−1^)	150	220	280
MgCl_2_6H_2_O (g L^−1^)	8	11	22.4
MgSO_4_7H_2_O (g L^−1^)	12	18	14.5
KCl (g L^−1^)	4	4	4
CaCl_2_ (g L^−1^)	1	1	1
NaHCO_3_ (g L^−1^)	0.5	0.5	0.5
Yeast extract (g L^−1^)	5	5	5
Agar (g L^−1^)	20	20	20
pH	8.2	7.9	7.7

The isolation of halophilic strains was performed from the TS5-SB culture by spreading 0.1 ml of serial dilution on Petri dishes in M3 agar medium and incubating at 37 °C for 7 days. Three colonies (S1, S2, and S3) were isolated in pure culture and then grown in 5 ml of M3 medium (shaking at 37 °C and 160 rpm for 7 days; Figure 3). These isolates were subjected to taxonomic and phylogenetic analyses.

#### 3.3.2 Genomic DNA extraction

Genomic DNA from halophilic strains (S1, S2, and S3) was isolated using the Wizard Genomic DNA Purification Kit. The genomic DNA was run on a 0.8% agarose gel in TAE buffer (40 mM Tris-acetate, 1 mM EDTA, pH 8.0) and visualized with a gel documentation system (Bio-Rad Laboratories, USA). The extracted DNA was stored at −20 °C until further use.

#### 3.3.3 PCR amplification

The amplification of the 16S rRNA gene from halophilic isolates (S1, S2, and S3) was performed using the archaeal-specific primer 21F (5′-TTCCGGTTGATCCYGCCGGA-3′) and the universal primer 1492R (5′-GGTTACCTTGTTACGACTT-3′). PCR reactions were carried out in a final volume of 50 μL containing 1.25 U of DreamTaq DNA Polymerase (Fermentas), 0.2 μM of each primer, 0.2 mM of each dNTP, and 1 × DreamTaq reaction buffer. The amplification program consisted of an initial denaturation at 94 °C for 4 min, followed by 30 cycles of denaturation at 94 °C for 1 min, annealing at 55 °C for 1 min, and extension at 72 °C for 1 min, with a final elongation step of 10 min at 72 °C. PCRs were performed using an Applied Biosystems 2720 Thermal Cycler (Life Technologies). The samples were analyzed by agarose (1.5%) gel electrophoresis and visualized on a gel documentation unit (Bio-Rad Laboratories, USA). The PCR products were purified using the Wizard SV Gel and PCR clean-up system (Promega Corporation) according to the manufacturer's protocol.

#### 3.3.4 Sequencing and phylogeny analysis

The nucleotide sequences of PCR products were determined using the Applied Biosystems SeqStudio Genetic Analyzer (CBS platform) with 21F and 1492R primers. Sequence similarity searches were performed using the online sequence analysis resources “BLAST” (Johnson et al., 2008). Multiple alignments were generated with the MUSCLE program (Edgar, 2004), and phylogenetic trees were constructed with the MEGA program version 12 (Kumar et al., 2024) based on evolutionary distances that were calculated by the Neighbor-Joining method (Saitou and Nei, 1987) according to the Jukes-Cantor model. The robustness of the inferred trees was evaluated by applying 100 bootstrap resamplings. The 16S rRNA ribosomal gene sequences for these isolates (S1, S2, and S3) have been deposited in GenBank under accession numbers PZ224748, PZ224749, and PZ224750, respectively.

#### 3.3.5 Experimental design

To evaluate the influence of halophilic microorganisms on salt crystallization, sixteen enrichment cultures were prepared in 250 ml Erlenmeyer flasks, each containing 100 mL of liquid medium M3. Cultures were inoculated with cell pellets from TS5-SB and TS5-BB precultures at inoculum ratios of 0.5, 1, 5, 10, 25, 50, 75, and 100%, corresponding to preculture volumes of 0.5, 1, 5, 10, 25, 50, 75, and 100 mL, respectively. Cell pellets were harvested by centrifugation (10 min, 5,000 rpm), quantified to determine the wet biomass weight of each inoculum, resuspended in 5 mL of medium, and used to inoculate the cultures under the same conditions as those applied for the enrichment cultures. The wet biomass concentration (mg mL^−1^) was calculated for each inoculum (Supplementary Table S3). The reported values in mg mL^−1^ correspond to fresh pellet mass per milliliter, calculated from the 5 mL volume of M3 medium used to resuspend each harvested pellet. Accordingly, eight inoculation levels were investigated for each brine origin, TS5-SB and TS5-BB.

A negative control containing sterile medium was also included. The cultures were incubated at 37 °C under continuous agitation (160 rpm) for 10–18 days, while the control was incubated for 45 days. After incubation, salt crystals were recovered by filtration, dried at 60 °C overnight, quantified, and subjected to morphological and mineralogical analysis. All cultures were conducted in duplicate.

The inoculated cultures were incubated for 21 days. During the incubation period, all cultures were regularly monitored for the formation of visible precipitates at the bottom of the flasks. The crystallization time endpoint was operationally defined as the first day on which visible precipitates were observed (Supplementary Table S3). For the abiotic control, incubation was extended until the first visible precipitation event, which occurred after 45 days.

#### 3.3.6 Microscopic observation

Cell morphology was subsequently examined using phase-contrast optical microscopy. Observations included cell shape, dimensions, and motility, providing the first characterization of the halophilic isolates obtained from the TS5-SB.

### 3.4 Characterization of precipitated salts

#### 3.4.1 Macroscopic observation of precipitated salts

Nine representative halite samples were selected for photographic analysis, including an abiotic control and a natural halite reference collected from solar saltern crystallization ponds. Seven biogenic halite samples derived from surface brine (SB) and bottom brine (BB) cultures across time and inoculum percentages were examined. One SB-0.5 and one BB-0.5 sample represented low inoculum conditions (0.5%). Intermediate conditions comprised 25% (SB-25) and 75% (BB-75) inocula, while high-inoculum conditions (100%) included one SB-100 and one BB-100 sample. An additional SB-10 sample at 10% inoculum was included, as it yielded the highest recovered salt precipitate mass and exhibited crystal morphology most consistent with the natural halite reference. Photographic documentation of all samples was performed under identical imaging conditions to allow consistent qualitative comparison of crystal size, habit, and textural organization across the full set of samples.

#### 3.4.2 Mineralogical characterization of precipitated salts by X-ray diffraction (XRD)

The nine representative salt precipitates (SB-0.5, SB-10, SB-25, SB-100, BB-0.5, BB-75, BB-100, T+, T–) were selected for mineralogical characterization by X-ray diffraction (XRD). This analysis was used to determine the mineral phases of all samples. Powder X-ray diffraction (XRD) analyses were conducted at the X-ray Diffraction Service Facility of the Faculty of Sciences of Tunis (FST), University of Tunis El Manar, using a PANalytical X'Pert Pro diffractometer equipped with Cu Kα radiation (λ = 1.5418 Å). Diffraction patterns were collected over a 2θ range of 6–60° at a scanning rate of 2° min^−1^.

#### 3.4.3 Morphological and elemental characterization by scanning electron microscopy (SEM–EDS)

The same nine representative samples analyzed by XRD were subsequently examined by scanning electron microscopy coupled with energy-dispersive X-ray spectroscopy (SEM–EDS) at the Research Center for Textile Science and Technology (CRSTT), University Campus of Monastir, Tunisia. Morphological investigation and elemental analysis of the precipitates were performed using a Thermo Scientific Phenom XL desktop scanning electron microscope equipped with a cerium hexaboride (CeB_6_) electron source, a backscattered electron detector (BSD), and an integrated EDS system. To preserve their pristine state, the salt crystals were examined without any chemical coating or pretreatment; individual precipitates were directly deposited and securely fixed onto the specimen holder prior to observation.

SEM imaging was conducted at an accelerating voltage ranging from 5.0 to 8.0 kV to thoroughly characterize crystal morphology, surface texture, aggregation features, and microstructural characteristics. Representative micrographs were acquired at different magnifications, with scale bars ranging from 50 to 200 μm across the analyzed fields of view. To avoid exhaustive chemical assumptions, EDS measurements were performed as point analyses on targeted areas of the crystals to obtain a semi-quantitative elemental composition and identify the major detectable elements associated with the precipitates. One or two representative analytical points were examined on selected crystals, and their corresponding EDS spectra were used to assess their elemental signatures.

### 3.5 Statistical analysis

All crystallization experiments were performed in duplicate (*n* = 2). For each condition, wet biomass (mg), wet biomass concentration (mg mL^−1^, based on a fixed 5 mL suspension), precipitated salt mass (g), crystallization duration (days), and crystallization rate (g day^−1^) were measured. Inoculum percentage (% v/v) was used only as a descriptor, whereas wet biomass introduced into the culture (mg) was used as the quantitative predictor. Crystallization rate was calculated as precipitated salt mass divided by crystallization duration. Data are reported as mean ± SD.

The effects of brine origin (surface brine, SB; bottom brine, BB) and wet biomass on salt precipitation and crystallization rate were assessed using ANCOVA, with brine origin as a fixed factor and wet biomass as a continuous covariate. Linear and quadratic biomass terms, including their interactions with brine origin, were evaluated to account for the observed non-linear response. Four nested models, ranging from a pooled model to fully origin-specific quadratic relationships, were compared using Akaike's Information Criterion (AIC); the model with the lowest AIC was retained for fitted curves and 95% confidence intervals. Models were weighted by the inverse variance (1/SD^2^).

Because SB and BB were compared at the same eight inoculum levels, paired differences were assessed using the paired *t*-test and Wilcoxon signed-rank test, with Welch's *t*-test used for pointwise comparisons at each level. Statistical significance was set at *p* < 0.05. Given the limited replication (*n* = 2), analyses were considered exploratory and interpreted alongside effect patterns and biological trends. All analyses were performed in Python using statsmodels and SciPy.

## 4 Results

### 4.1 Geochemical characterization of brines from Sfax

#### 4.1.1 Evolution of physicochemical parameters

Available data on water temperature in the Sfax solar salterns indicate a strong correlation with regional atmospheric temperatures, ranging from 24 °C to 34 °C during the warm season. Consequently, brine density, which is governed by the interplay of salinity, temperature, and hydrostatic pressure, increases progressively from 1,026 in seawater inlet basins to 1,347 in magnesium-rich brine ponds, with salinity being the dominant factor. Up to a density of 1,215, this increase is approximately linear with the concentration factor; however, beyond this point, evaporation and mineral precipitation become the primary drivers of density changes (Figure 4B).

**Figure 4:**
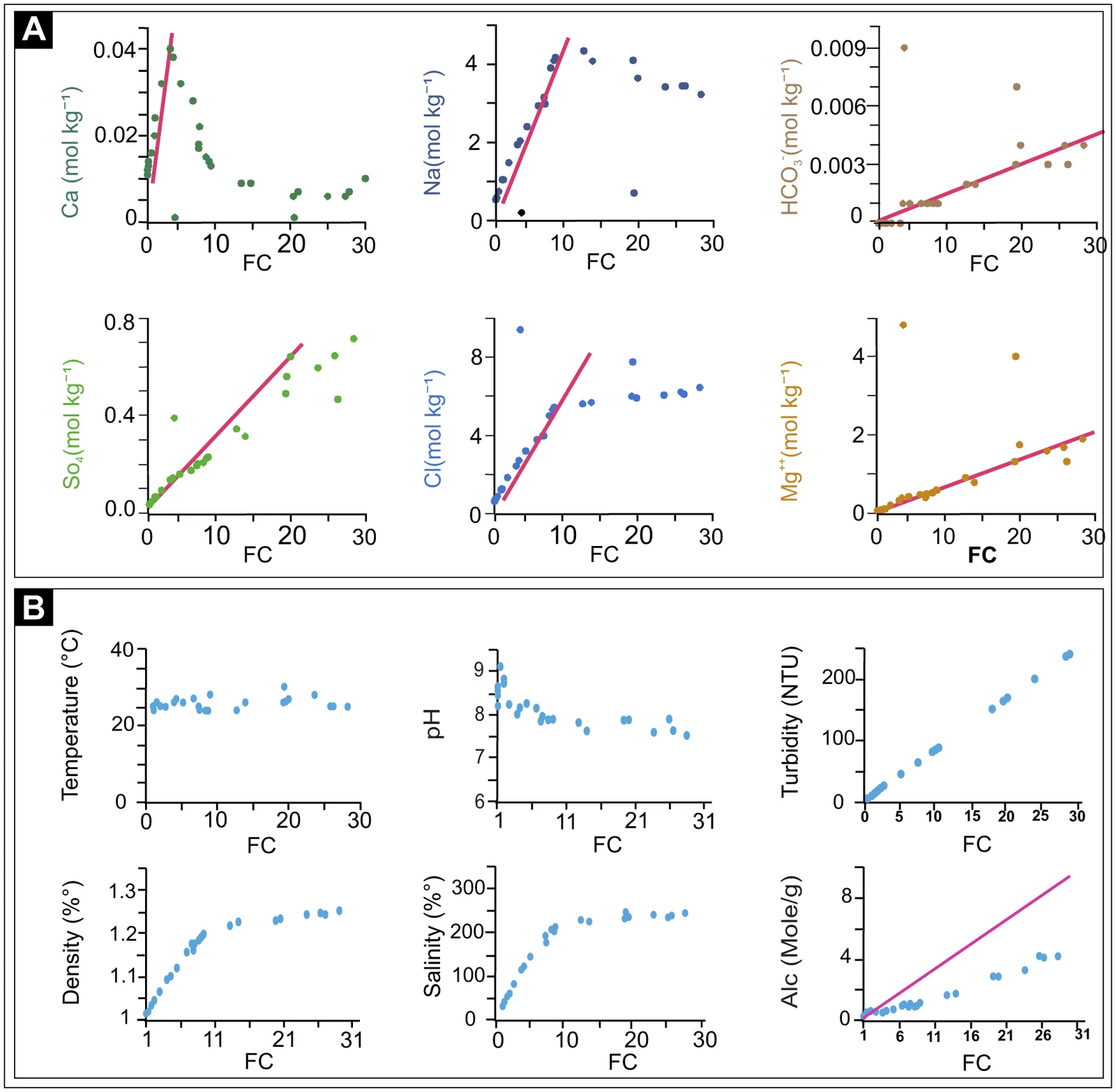
Geochemical characterization of the 26 sampled brines: **(A)** evolution of major ion concentrations during the evaporitic process, expressed in mol kg^−1^, and **(B)** evolution of physicochemical parameters during evaporation. The red line represents the evaporation curve.

Similarly, salinity follows a comparable trend, rising in response to evaporation and salt precipitation. Regarding pH, it gradually declines from 9.2 to 7.6 along the brine pathway, ultimately reaching around 6.1 in the most concentrated solutions (Figure 4B).

#### 4.1.2 Evolution of major ions

Calcium carbonate precipitates early during the evaporation process, either through chemical reactions or microbial activity; however, this initial precipitation has little effect on the Ca^2+^ levels in the Sfax solar saltern brines. Indeed, calcium concentrations maintain a linear relationship with the potassium-based concentration factor (FC) until reaching approximately five times the initial seawater concentration. Subsequently, as gypsum precipitation intensifies (FC = 5–8), calcium levels decline sharply, after which they decrease more gradually and eventually stabilize in the most concentrated brines, marking the completion of gypsum deposition.

In contrast, sulfate concentrations increase steadily with FC, primarily driven by evaporation rather than gypsum precipitation, which largely compensates for sulfate losses. Initially, a slight dip in sulfate is observed when gypsum formation begins (FC = 5); nevertheless, this effect is quickly overridden by ongoing evaporation (Figure 4A). Sulfate levels continue to rise until the brine reaches saturation with sulfate salt in the crystallizer ponds.

Meanwhile, in the early ponds (salinity < 200‰), sodium and chloride concentrations increase linearly with FC as a direct consequence of evaporation. Although halite formation starts around FC ≈ 11 (salinity ~260‰), NaCl precipitation remains minimal, preserving the linear trend. However, once FC reaches 13–17 (Figure 4A), the brines become saturated with halite, leading to rapid Na^+^ precipitation and a sharp decrease in its concentration relative to Cl^−^. Chloride continues to rise more gradually, effectively compensating for the loss of K^+^ and Mg^2+^ ions during halite deposition.

Finally, magnesium concentrations increase continuously with FC, reflecting progressive evaporation. magnesium. Although it can be incorporated into evaporite minerals at early stages, this trend becomes significant only after extensive salt precipitation in the crystallizer ponds (FC ~25; Figure 4A). Magnesium's contribution to evaporite formation is delayed due to its initially low concentration and the high solubility of magnesium salts compared with gypsum, sodium, and potassium chlorides.

Piper diagrams (Supplementary Figure S1), commonly used in hydrogeology (Piper and Garrett, 1953), help classify water types and understand hydrochemical facies by analyzing the concentrations of Ca^2+^, Mg^2+^, Na^+^, K^+^, ${\text{HCO}}_{3}^{-}$, Cl^−^, and ${\text{SO}}_{4}^{2-}$. The analysis reveals that chloride (Cl^−^) is the dominant anion in the samples, while the cations fall into two main groups: monovalent (Na^+^, K^+^) and divalent (Ca^2+^, Mg^2+^), showing clear hydrochemical differences. The identified water types include Na-Cl and Na-Ca-Cl, with Na-Cl being the most prevalent in hypersaline waters.

### 4.2 Microbial community dynamics and functional contributions to halite crystallization in the Sfax solar salterns

#### 4.2.1 Isolation and molecular characterization of halophilic archaea

To investigate the microbial communities across the Sfax solar salterns, three representative ponds spanning the salinity gradient, M22 (moderate salinity), B22 (high salinity), and TS5 (hypersaline), were selected based on their distinct geochemical characteristics (Table 1, Supplementary Figure S2). Total salinity increased progressively from 186.206 to 364.560 g L^−1^, accompanied by a shift in major-ion composition, notably a decrease in Ca^2+^ and increases in Mg^2+^ and K^+^. Surface brine (SB) and bottom brine (BB) samples from each pond were used to inoculate enrichment cultures containing 150, 220, and 280 g L^−1^ NaCl, corresponding approximately to the salinity ranges of the three representative ponds (Table 1, Supplementary Table S1, Supplementary Figure S2). Six enrichment culture conditions were established (M22-SB, M22-BB, B22-SB, B22-BB, TS5-SB, and TS5-BB; Supplementary Figure S3A).

In the M3 medium containing 280 g/L of salt (TS5-SB and TS5-BB; Supplementary Figure S2B), aerobic microorganisms developed a red pigmentation after 4–7 days of incubation, while cultures inoculated with sediment samples showed a two-day delay in pigment formation. In the M2 medium (B22-SB and B22-BB), the red coloration appeared after 7–10 days, whereas cultures in the M1 medium (M22-SB and M22-BB) remained yellow throughout the experiment. From the second week onward, salt crystals were abundantly present in the culture medium of the M3 flasks, appeared later in the B22-SB and B22-BB cultures, and were absent in the M22-SB, M22-BB, and negative control cultures.

From the TS5-SB enrichment culture, three extremely halophilic archaeal isolates (designated S1, S2, and S3) capable of growing at 28% (w/v) NaCl were successfully obtained. After 4 days of incubation, microscopic observations revealed elongated, slender rod-shaped cells measuring 5–10 μm in length (Figure 5B).

**Figure 5:**
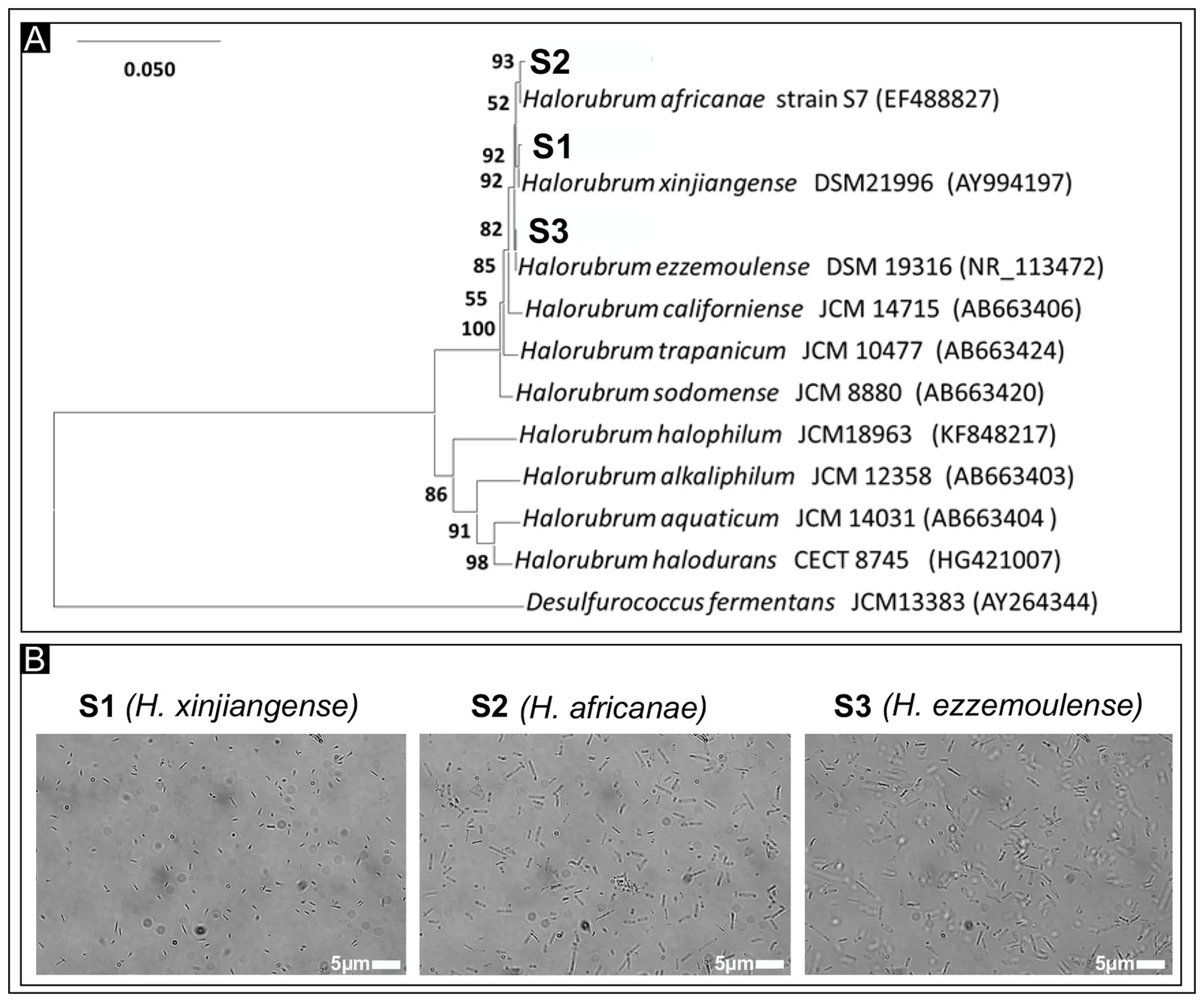
**(A)** Phylogenetic tree based on 16S rRNA gene sequence similarities between the archaeal isolates and their closest relatives. The tree was constructed using the Jukes–Cantor model and the Neighbor–Joining method. *Desulfurococcus fermentans* was used as the outgroup. Bootstrap values are based on 100 replicates. **(B)** Phase-contrast micrographs of representative *Halorubrum* strains (S1 = *H. xinjiangense*, S2 = *H. africanae*, S3 = *H. ezzemoulense*) isolated from aerobic enrichment culture TS5-SB in the Sfax solar salterns.

The phylogenetic affiliations of the isolates were determined based on 16S rRNA gene sequence analysis. Phylogenetic trees depicting the relationships between the isolates and their closest relatives are shown in Figure 5A. All isolates were assigned to the phylum *Euryarchaeota* and the family *Haloferacaceae*, within the genus *Halorubrum*. Isolates S1, S2, and S3 exhibited 99.85%, 99.71%, and 100% sequence similarity with *Halorubrum xinjiangense, Halorubrum africanae*, and *Halorubrum ezzemoulense*, respectively. These results suggest that the genus *Halorubrum* was the dominant genus in the Sfax saltern ponds at high salinity (280 g/L).

#### 4.2.2 Microbial influence on halite nucleation and precipitation

To investigate the dynamics of microbially mediated crystallization, we examined the effects of inoculum origin and biomass concentration on halite precipitate mass and crystallization kinetics in SB and BB cultures (Figure 6). The results revealed a clear influence of inoculum origin on crystallization performance. SB cultures generally produced more precipitated salt and showed higher crystallization rates than BB cultures across the different inoculum levels. The only exception was BB-1, which showed slightly higher recovered salt precipitate mass and crystallization rate than SB-1. Overall, these results indicate that the surface-brine inoculum generally promoted greater salt precipitation and faster crystallization than the bottom-brine inoculum.

**Figure 6:**
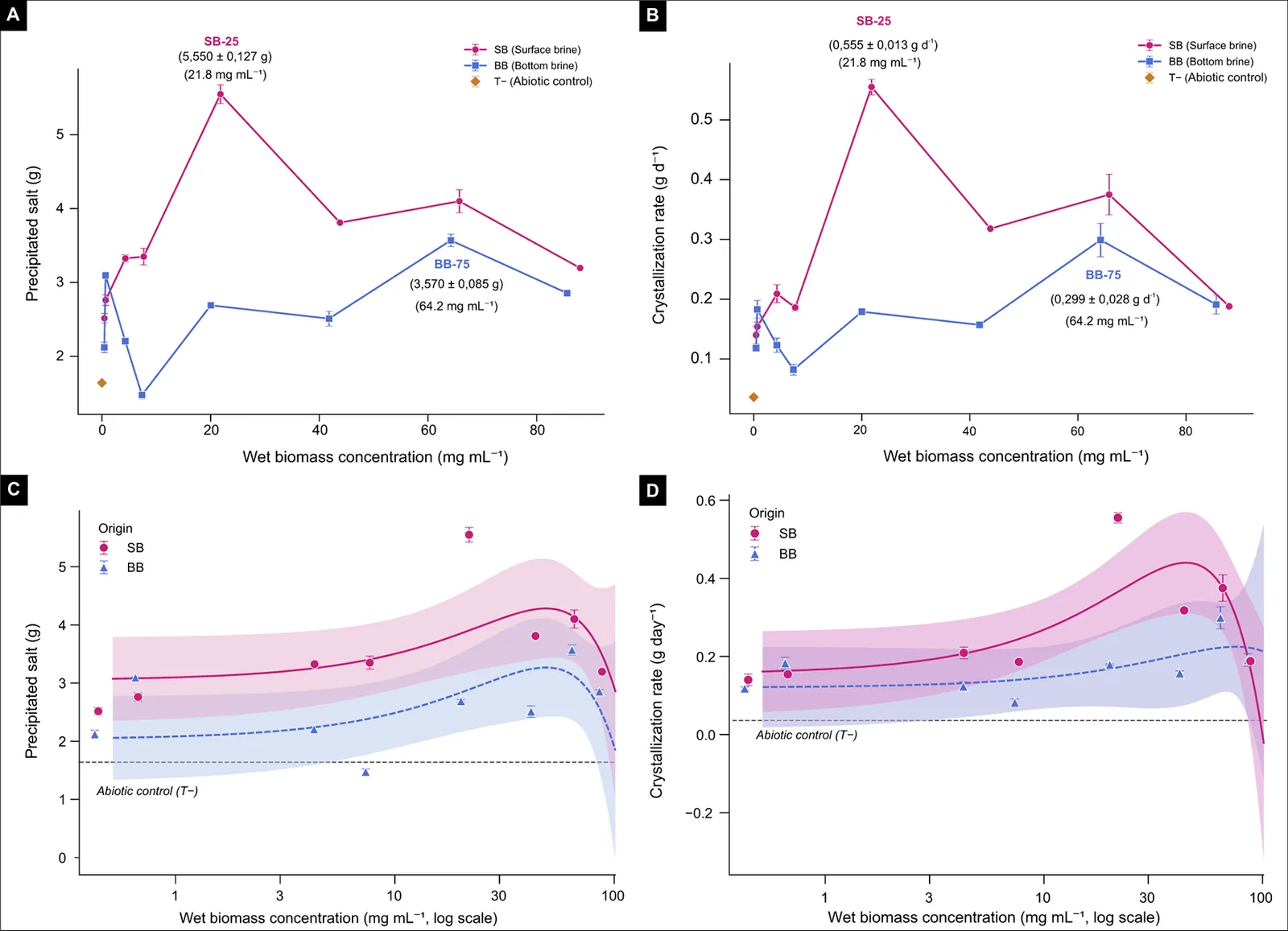
Statistical and experimental assessment of biomass-dependent halite precipitation and crystallization in surface- and bottom-brine enrichment cultures. **(A)** Experimental variation in halite precipitation as a function of wet biomass concentration in surface- (SB) and bottom-brine (BB) cultures; and **(B)** corresponding variation in crystallization rate. **(C)** ANCOVA-based assessment of the effects of wet biomass concentration, brine origin, and their interaction on halite precipitation; **(D)** corresponding ANCOVA results for crystallization rate; Curves connect the experimental mean values for visualization purposes only and do not represent fitted statistical models. Error bars represent standard deviations (SD); T^−^ denotes the uninoculated abiotic control.

The response to increasing biomass concentration was distinctly non-linear in both inoculum types (Figures 6A, B). In SB cultures, the recovered salt precipitate mass increased progressively with increasing inoculum and reached a maximum at 25%, corresponding to a wet biomass concentration of 21.80 ± 0.894 mg mL^−1^. Under this condition, 5.550 ± 0.127 g of salt was precipitated within 10 days, giving the highest crystallization rate recorded for SB (0.555 ± 0.013 g d^−1^). Further increases in biomass concentration did not enhance salt precipitation but instead resulted in a decline in halite-dominated precipitate mass at higher inoculum concentrations. BB cultures displayed a different response pattern (Figures 6C, D). Halite-dominated precipitate mass initially increased at low inoculum concentrations, declined at intermediate levels, and subsequently increased again at higher biomass concentrations. The maximum recovered salt precipitate mass was observed at 75% inoculum, corresponding to a wet biomass concentration of 64.20 ± 2.632 mg mL^−1^. This treatment yielded 3.570 ± 0.085 g of salt after 12 days, with a crystallization rate of 0.299 ± 0.028 g d^−1^. Overall, SB reached its maximum recovered salt precipitate mass at a substantially lower biomass concentration than BB.

The difference between the two inoculum origins was particularly pronounced when crystallization kinetics were considered (Figure 6). The maximum crystallization rate observed with SB was approximately twice as high as that recorded for BB. Furthermore, at the common inoculum level of 25%, the crystallization rate for SB was approximately three times higher than that for BB. These findings indicate that the effect of inoculum origin was not limited to the final yield of precipitated salt but also influenced the kinetics of crystallization, with SB promoting a more rapid conversion of the hypersaline medium into crystalline material.

A large difference was also observed between inoculated cultures and the abiotic control. The uninoculated treatment (T–) produced only 1.640 ± 0.014 g of salt over 45 days, corresponding to a crystallization rate of only 0.036 ± 0.001 g d^−1^. Compared with the abiotic control, SB-25 produced more than three times as much precipitated salt and reached crystallization substantially faster (Figure 6). Its operational crystallization rate was substantially higher than that of the abiotic control. These results suggest that the presence of the microbial consortium enhanced both the extent and the kinetics of salt precipitation under experimental conditions.

#### 4.2.3 Crystal morphology at macroscopic and microscopic scale

At the end of the crystallization experiments, the precipitates recovered from the enrichment cultures were characterized at macroscopic and microscopic scales using macroscopic imaging, scanning electron microscopy (SEM), and energy-dispersive X-ray spectroscopy (EDS; Figure 7). The precipitates predominantly consisted of cubic to subcubic crystals consistent with halite, although marked differences were observed in crystal size, degree of aggregation, faceting, surface texture, and population homogeneity among the experimental conditions.

**Figure 7:**
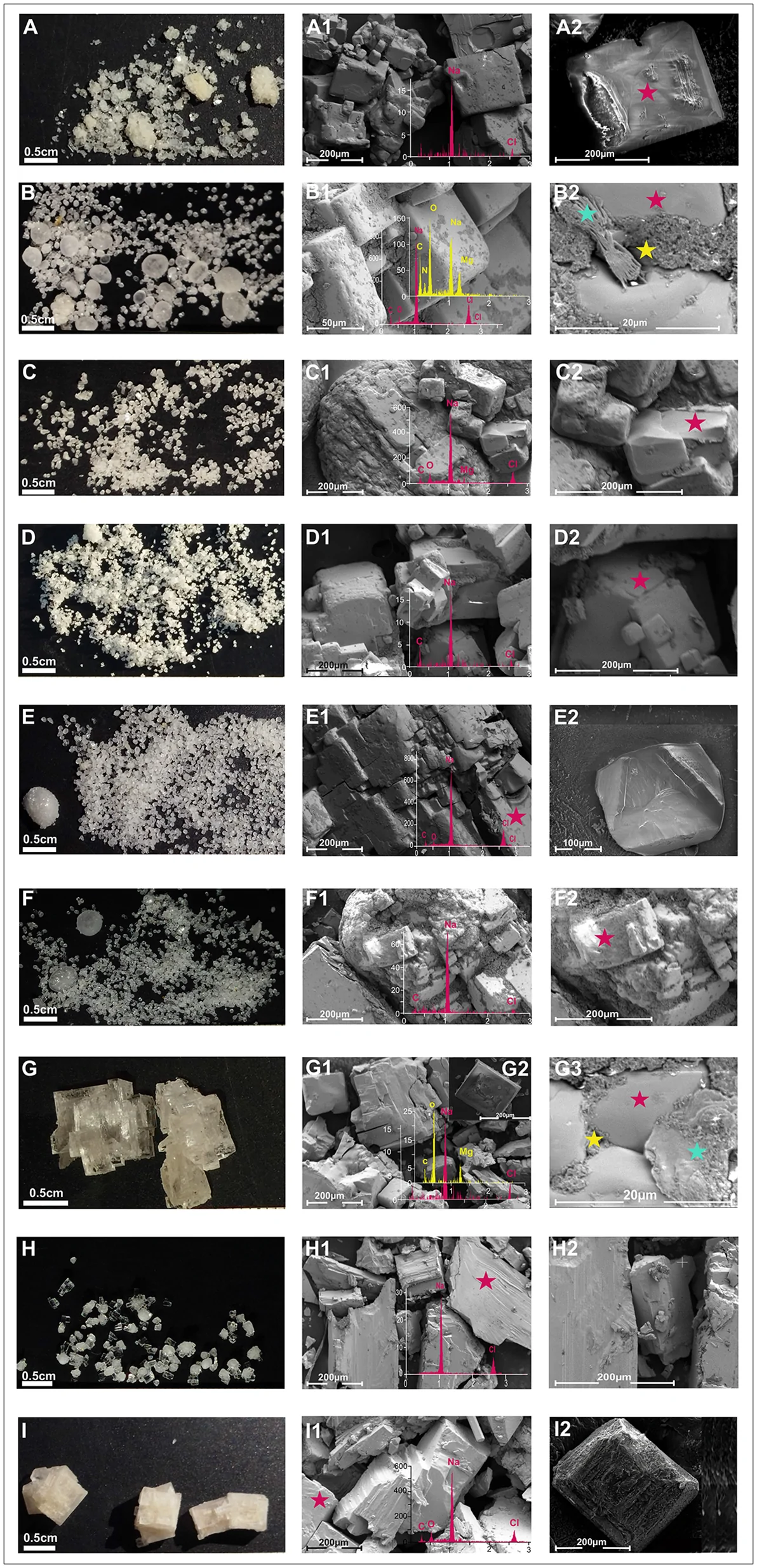
Representative macroscopic photographs, scanning electron microscopy (SEM) micrographs, and energy-dispersive X-ray spectroscopy (EDS) analyses of nine halite samples recovered from salt crystallization experiments conducted with increasing wet-biomass inocula in surface brine (SB) and bottom brine (BB) cultures, together with the natural halite reference (T^+^) and abiotic control (T^−^). SB-0.5 **(A–A2)** shows macroscopic salt aggregates **(A)**, SEM evidence of crystal rearrangement and aggregation **(A1)**, and an individual crystal with a cubic morphology **(A2)**. SB-25 **(B–B2)** displays macroscopic precipitates **(B)**, well-associated crystals forming aggregates while retaining distinct crystal boundaries **(B1)**, and halite crystals associated with gypsum sheets and C- and N-bearing material potentially associated with organic matter **(B2)**. SB-100 **(C–C2)** shows macroscopic salt crystals **(C)**, the corresponding SEM crystal population **(C1)**, and enlarged crystals with poorly defined edges and irregular morphologies **(C2)**. BB-0.5 **(D–D2)** is characterized by macroscopic salt precipitates **(D)**, SEM evidence of crystal rearrangement and aggregation **(D1)**, and a heterogeneous population comprising small- to medium-sized crystals **(D2)**. BB-75 **(E–E2)** displays macroscopic crystals **(E)**, closely associated crystals forming aggregates **(E1)**, and an enlarged, relatively well-developed individual crystal **(E2)**. BB-100 **(F–F2)** shows macroscopic precipitates **(F)**, crystal rearrangement and aggregation **(F1)**, and crystals with poorly defined and irregular morphologies **(F2)**. T^+^ **(G–G3)** comprises macroscopic aggregates of natural halite from the Sfax saltern **(G)**, the corresponding SEM crystal population **(G1)**, a well-developed individual cubic crystal **(G2)**, and crystal aggregates containing organic-rich material and gypsum sheets coated with organic material **(G3)**. T^−^ **(H–H2)** shows macroscopic precipitates **(H)**, a heterogeneous SEM crystal population **(H1)**, and enlarged crystals displaying poorly defined morphologies **(H2)**. SB-10 **(I–I2)** displays macroscopic salt aggregates **(I)**, the corresponding SEM crystal population **(I1)**, and an individual cubic crystal with well-defined edges **(I2)**. Colored stars indicate representative areas selected for EDS analysis: red–pink and yellow stars mark EDS analysis sites, with yellow stars highlighting organic-rich material, whereas light-blue stars identify gypsum sheets. Scale bars: 0.5 cm for macroscopic photographs and 200–20 μm for SEM micrographs.

Under pre-optimal biomass conditions, SB-0.5 and BB-0.5 exhibited small precipitate aggregates. SEM observations revealed heterogeneous populations dominated by fine, closely associated crystals, with occasionally larger individuals. SB-0.5 locally displayed truncated cubic crystals with irregular faces and edges, whereas BB-0.5 contained small to medium-sized crystals with variable degrees of individualization and attachment. EDS spectra were mainly dominated by Na and Cl, confirming the halitic composition of the analyzed crystalline domains.

Under optimal biomass conditions, SB-25 and BB-75 produced more abundant precipitates and larger, better-developed crystals. SB-25 was characterized by a relatively homogeneous population of well-faceted cubic crystals with planar faces, sharp edges, and clearly defined boundaries. Locally, halite crystals were associated with gypsum sheets and C- and N-bearing material potentially associated with organic matter. The latter showed C, N, O, Na, and Mg by EDS, whereas the adjacent crystalline domains were dominated by Na and Cl. Although this C–N enrichment is consistent with the presence of C- and N-bearing material potentially associated with organic matter, its specific nature and whether it corresponds to extracellular polymeric substances (EPS) cannot be determined from EDS alone. BB-75 also showed relatively large and well-developed crystals, but with stronger crystal attachment and intergrowth. Overall, SB-25 exhibited the most homogeneous and well-faceted crystal population among the experimental treatments.

Under post-optimal biomass conditions, SB-100 and BB-100 displayed greater morphological heterogeneity, with numerous aggregates and crystals of variable size. SEM observations showed cubic to subcubic crystals with less regular outlines, less-defined edges, and locally irregular surface textures, accompanied by pronounced crystal association. Their EDS signatures varied locally, but crystalline domains were generally characterized by Na and Cl, with additional C, O, and Mg detected in some areas.

The natural halite reference (T^+^) consisted of larger, well-individualized cubic crystals with planar faces and sharp edges. Halite was locally associated with gypsum and organic-rich material, similarly to the experimental SB-25 precipitates. In contrast, the abiotic control (T^−^) showed smaller, more heterogeneous aggregates with less clearly defined crystal outlines. Its EDS signature was mainly dominated by Na and Cl, providing a reference for halite precipitation in the absence of biomass.

Finally, SB-10 exhibited an intermediate morphology between the pre-optimal and optimal conditions. Its crystals were more developed and individualized than those of SB-0.5 but less homogeneous and well-faceted than those of SB-25. Some crystals displayed concentric growth steps while retaining an overall cubic habit. Their EDS spectra were dominated by Na and Cl, confirming their halitic composition. Overall, the combined SEM-EDS observations indicate that biomass level strongly influenced halite crystal development, aggregation, and surface morphology, with the most ordered and well-faceted crystals occurring under optimal biomass conditions.

#### 4.2.4 Mineralogical composition and phase identification of precipitated salts

To identify and confirm the mineral phases, nine representative salt precipitate samples (SB-0.5, SB-10, SB-25, SB-100, BB-0.5, BB-75, BB-100, T+, and T–) were subjected to mineralogical characterization by XRD. All XRD patterns showed a clear dominance of halite (NaCl) in all nine analyzed samples, regardless of brine origin (SB or BB) and biomass level (Figure 8). The biogenic precipitates exhibited the characteristic reflections of cubic halite at *d* ≈ 3.25, 2.81, 1.99, 1.70, and 1.62 Å, closely matching those of both the abiotic control (T^−^) and the natural halite reference (T^+^). This consistent correspondence confirms that halite was the predominant crystalline phase in all precipitates.

**Figure 8:**
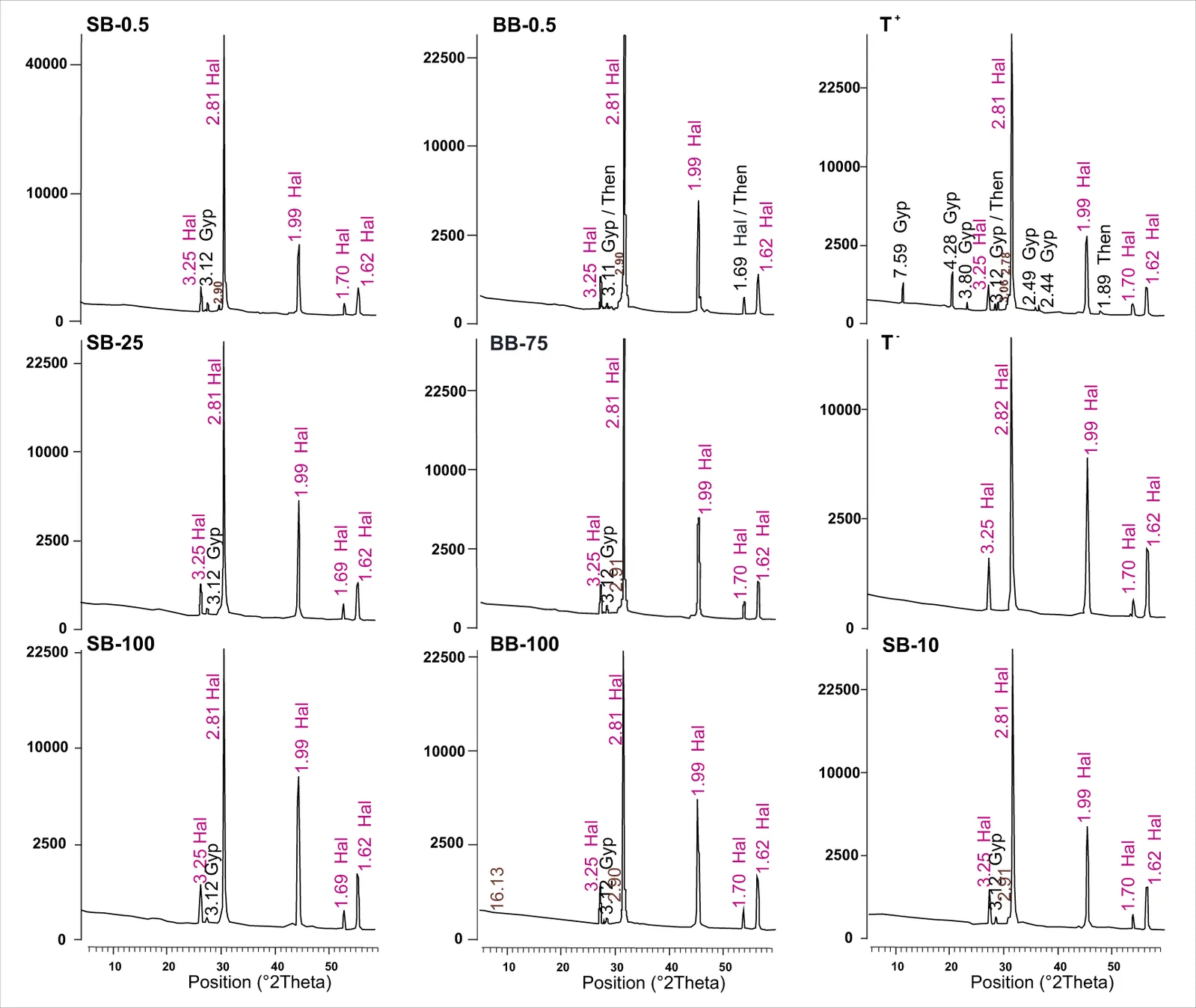
Powder X-ray diffraction (XRD) patterns of representative crystals obtained during salt crystallization experiments performed with increasing concentrations of wet biomass as inoculum in bottom brine (BB) and surface brine (SB) cultures. Hal, halite; Gyp, gypsum; Then, thenardite. The nine salt precipitate samples analyzed were SB-0.5, SB-10, SB-25, SB-100, BB-0.5, BB-75, and BB-100. T^+^, natural halite reference; T^−^, abiotic control.

Minor accessory sulfate phases were detected in several samples. Weak gypsum reflections were observed in SB-0.5, SB-10, SB-25, SB-100, BB-75, and BB-100, whereas BB-0.5 additionally showed weak reflections tentatively attributed to gypsum/thenardite and thenardite (Figure 8). The natural reference (T^+^) displayed a more diverse set of gypsum reflections together with a thenardite reflection, indicating the natural occurrence of minor sulfate phases associated with halite crystallization.

The comparison between T^−^ and T^+^ revealed a clear mineralogical distinction. While T^−^ was essentially characterized by halite, T^+^ contained several additional gypsum and thenardite reflections. The occurrence of similar minor sulfate phases in some biogenic precipitates therefore cannot be uniquely attributed to microbial activity but may reflect the natural mineralogical complexity of the brine and/or the coprecipitation of accessory phases during halite formation.

An additional reflection at *d* = 16.13 Å was detected in BB-100 but remained unidentified, while several weak reflections in the *d* ≈ 2.78–3.06 Å range were considered insufficiently diagnostic for reliable mineral assignment.

## 5 Discussion

### 5.1 Evaporative brine evolution and geochemical drivers of sequential evaporite formation in the Sfax solar saltern system

Monitoring the physicochemical parameters of saline ponds is essential to track salt production dynamics and understand the controls on mineral precipitation (Javor, 2006; Babel and Schreiber, 2014; Konstantinou et al., 2023). Geochemical analysis of 26 brine samples from the Sfax solar salterns confirmed marine-fed evaporative sequences (Figure 4, Supplementary Figure S1, Supplementary Table S1), in which carbonate precipitates first, followed by gypsum, halite, and, in the final stages, K–Mg salts. These data provide a framework for interpreting mineral evolution and assessing the influence of pond-specific conditions on sequential evaporite formation. This sequence is consistent with the classical evolutionary pathway described for marine evaporative systems and broadly resembles the mineralogical succession observed in natural saline environments, including sebkhas and chotts of North Africa and the Middle East (Hakdaoui et al., 2020; Zedam et al., 2022).

This trajectory is reflected in the Piper diagram (Piper and Garrett, 1953; Supplementary Figure S1), where the progressive displacement of brine signatures from Ca–SO_4_/HCO_3_ facies toward a distinctly Na–Cl–dominated field in late-stage brines demonstrates a systematic ionic restructuring driven by evaporative concentration and sequential mineral saturation. Thus, increasing evaporation and salinity are accompanied by a lateral evolution of sedimentary and geochemical facies, from relatively Ca–SO_4_/HCO_3_-rich conditions toward increasingly Na–Cl-dominated and, ultimately, highly concentrated brines.

Correspondingly, the delayed increase in Mg^2+^ abundance at high salinity aligns with the expected exclusion of Mg during earlier Ca-dominated precipitation and suggests efficient pond staging and residence-time control within the saltern system (Ghadimi and Azadifar, 2023).

This salinity-driven evolution of facies is accompanied by a corresponding spatial differentiation of microbial communities (Figure 9, Supplementary Table S2). Salinity acts as a major ecological filter in hypersaline environments, selecting microbial populations according to their tolerance to increasing osmotic stress (Oren, 2002, 2013). Thus, increasing salinity may promote a shift from moderately halophilic to highly and extremely halophilic communities, encompassing diverse microbial groups (Ventosa et al., 2015). The saltern therefore represents not only a geochemical and mineralogical gradient but also an ecological gradient shaping microbial community composition and diversity.

**Figure 9:**
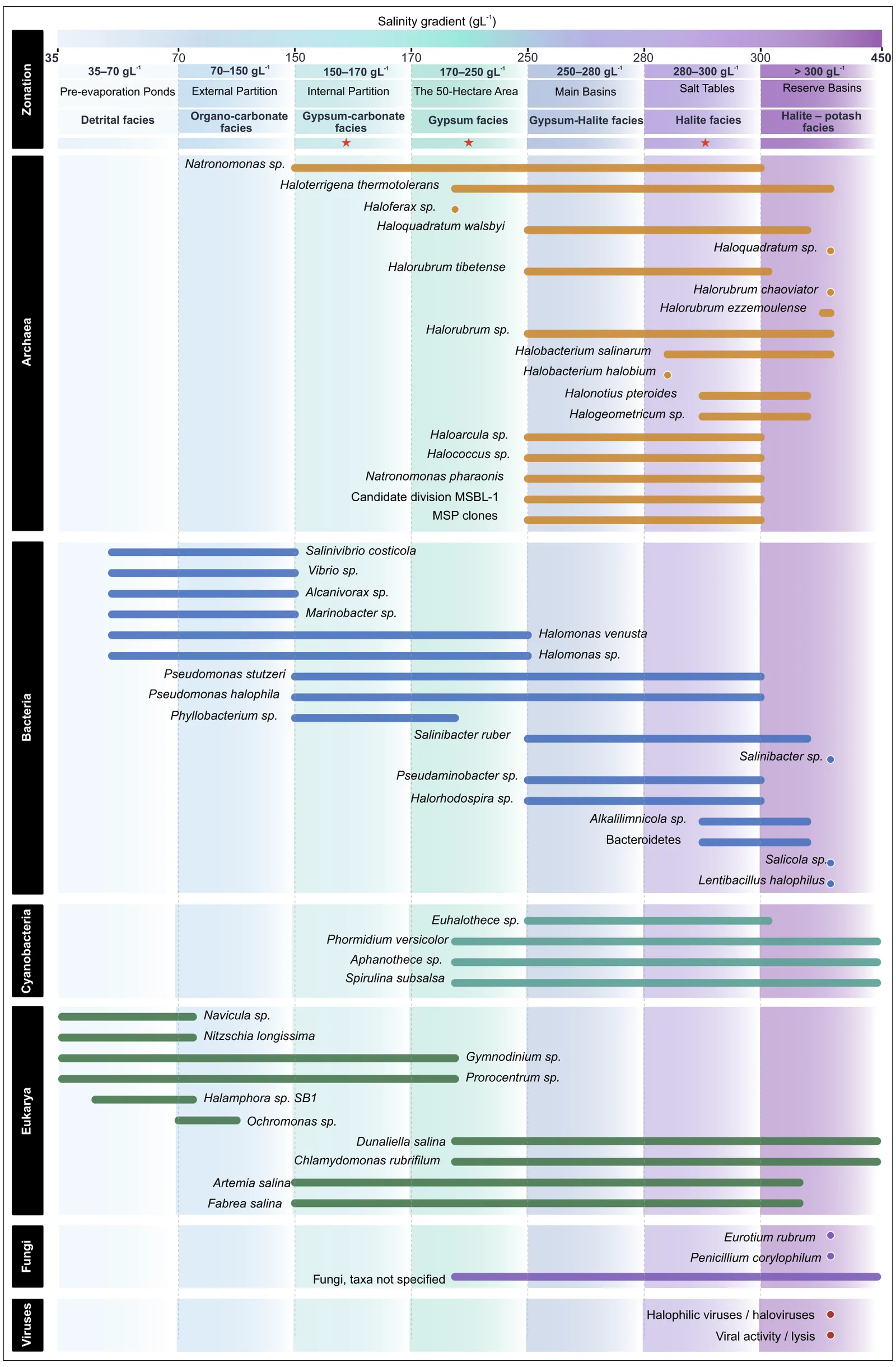
Salinity-driven microbial-sedimentary zonation in the Sfax Solar Saltern, Tunisia. The salinity gradient and the associated microbial-sedimentary facies succession were reconstructed primarily from the geochemical and sedimentological framework established by Amdouni (1990) for the Sfax solar saltern, complemented by data compiled and adapted from published sources (Supplementary Table S2). The M22, B22, and TS5 brine samples represent different salinity and geochemical conditions along the evaporation gradient, with red stars marking their sampling ponds.

Although the overall evolution fits established evaporite models, minor discrepancies between ponds indicate basin-scale hydrodynamic variability, episodic recharge, and subtle operational differences, which mirror observations reported from comparable sebkha and chott systems in North Africa and the Middle East (Hakdaoui et al., 2020; Zedam et al., 2022).

### 5.2 Isolation of halophilic archaeal strains from the saline of Sfax

Halophilic microorganisms are key components of saline and hypersaline ecosystems, shaping biogeochemical processes and responding sensitively to physicochemical fluctuations (Des Marais, 2003; Pace et al., 2016; Al Disi et al., 2023). Their distribution is largely controlled by salinity gradients, which affect both community structure and functional potential (Oren, 2024). In this study, enrichment strategies mimicking the three major salinity regimes of the Sfax solar salterns effectively selected taxa adapted to specific ionic conditions. Three archaeal strains were successfully isolated from the most salinity-concentrated enrichment medium (TS5-SB, 280 g/l NaCl) and developed intense red pigmentation, characteristic of carotenoid-rich, extremely halophilic microorganisms. This chromatic shift not only indicated their dominance under extreme salinity but also reflected physiological responses to osmotic and photic stress, which increase with salinity.

Phylogenetic analyses revealed that isolates S1, S2, and S3 were affiliated with *Halorubrum xinjiangense, Halorubrum africanae*, and *Halorubrum ezzemoulense*, respectively. Members of the genus *Halorubrum* have been frequently reported from hypersaline habitats worldwide (Feng et al., 2004; Kharroub et al., 2006; Najjari et al., 2021; Han et al., 2022). *Halorubrum* is one of the largest genera within the class Halobacteria and comprises predominantly aerobic, Gram-negative, chemoorganotrophic archaea (Parte et al., 2020; Oren, 2020). Members of this genus have been isolated from a wide range of saline environments, including salt lakes, saline soils, and solar salterns (Amoozegar et al., 2017; Han et al., 2022; Vreeland et al., 2023), reflecting their broad ecological distribution and remarkable adaptation to hypersaline conditions.

Beyond their taxonomic significance, members of *Halorubrum* may play important roles in hypersaline ecosystems through the production of carotenoid pigments, synthesis of extracellular polymeric substances (EPS), and formation of structured biofilms. These physiological traits may promote surface adhesion and microbial aggregation while contributing to the stabilization of local microenvironments under extreme salinity (Oren, 2002; Antón et al., 2002; Amoozegar et al., 2017).

Comparative evidence from isolates recovered from solar salterns in Portugal, China, Mexico, and the Philippines indicates that *Halorubrum* species exhibit broadly comparable physiological characteristics and adaptive responses to high salinity and osmotic stress (Albuquerque et al., 2024; Yin J. et al., 2015; Yin S. et al., 2015; Vázquez-Madrigal et al., 2021; Han et al., 2022). These recurrent adaptive features across geographically distinct hypersaline environments suggest that similar environmental constraints may impose strong selective pressures, favoring the persistence of archaeal lineages with conserved mechanisms for coping with extreme salinity.

The isolation of three strains, *Halorubrum xinjiangense, Halorubrum africanae*, and *Halorubrum ezzemoulense*, from the TS5-SB enrichment cultures indicates a marked enrichment of this archaeal lineage under the cultivation conditions applied and further supports its occurrence in the crystallization ponds of the Sfax solar salterns. Previous studies conducted in the Sfax salterns have likewise reported the isolation and identification of *Halorubrum tibetense* (Baati et al., 2008), *Halorubrum chaoviator* (Ghanmi et al., 2016, 2020), *Halorubrum ezzemoulense* (Frikha-Dammak et al., 2016), and other *Halorubrum* spp. (Baati et al., 2008; Trigui et al., 2011; Ghanmi et al., 2016, 2020) from hypersaline ponds, particularly the TS crystallizers (Figure 9, Supplementary Table S2). These findings further corroborate the recurrent occurrence of *Halorubrum* in the highly saline crystallization ponds of the Sfax salterns.

The enrichment and persistence of *Halorubrum* in Sfax brines likely reflect its adaptation to extreme salinity and may contribute to saltern ecosystem functioning through EPS production, microbial interactions, and modification of mineral–microbe interfaces, potentially influencing halite nucleation and growth. However, its direct role in halite biomineralization remains to be experimentally demonstrated.

### 5.3 Microbially influenced mineral precipitation and crystal structure

Hypersaline environments represent ideal model systems for investigating the interplay between geochemical processes and microbial activity, where evaporite formation reflects a tight coupling between evaporative concentration, ionic supersaturation, mineral nucleation, and microbial dynamics (Oren, 2002; Dupraz et al., 2009; Warren, 2016). In solar salterns, evaporation is the primary physicochemical process driving salt precipitation by progressively concentrating dissolved ions until supersaturation is reached. However, once the thermodynamic conditions for halite precipitation are established, microbial community structure, metabolic activity, and environmental conditions may influence the efficiency and kinetics of nucleation and crystal growth. In these extreme ecosystems, halite biomineralization cannot be interpreted merely as a physicochemical process dictated by solubility equilibria, but rather as an emergent property shaped by microbial community structure, metabolic activity, and environmental constraints (Visscher and Stolz, 2005; Pedone et al., 2020; Tostevin and Poulton, 2023). To investigate this interaction, we assessed whether increasing microbial biomass in TS5-SB and TS5-BB enrichment cultures influenced halite crystallization, thereby providing an experimental framework for evaluating the potential contribution of enriched halophilic communities to mineral formation under hypersaline conditions. Our findings show that microbial enrichment substantially accelerated halite precipitation relative to the abiotic control, whereas the response to increasing biomass was distinctly nonlinear and differed according to inoculum origin (Figure 6).

#### 5.3.1 Triphasic biomass-dependent modulation of halite crystallization

Our results demonstrate that microbial biomass substantially enhanced halite crystallization compared with the abiotic control. Under abiotic conditions, only 1.640 ± 0.014 g of salt precipitated over approximately 45 days, corresponding to a crystallization rate of 0.036 ± 0.001 g day^−1^ (Supplementary Table S3). In contrast, enriched microbial cultures produced considerably more salt over shorter incubation periods. The most efficient treatment, SB-25, produced 5.550 ± 0.127 g of salt after only 10 days, corresponding to an operational crystallization rate of 0.555 ± 0.013 g day^−1^. This rate was substantially higher than that of the abiotic control. The maximum recovered salt precipitate mass in the BB series was observed at 75% inoculum, corresponding to a wet biomass concentration of 64.20 ± 2.632 mg mL^−1^. This treatment yielded 3.570 ± 0.085 g of salt after 12 days, with a crystallization rate of 0.299 ± 0.028 g day^−1^ (Figure 6, Supplementary Table S3).

These results confirm that evaporation remains the fundamental physicochemical driver of halite precipitation by progressively concentrating dissolved Na^+^ and Cl^−^ until supersaturation is reached, whereas the marked increase in crystallization rate in the presence of microbial biomass suggests that microorganisms can substantially accelerate and modulate the precipitation process. This effect is consistent with the potential contribution of microbial cells and extracellular polymeric substances (EPS), which may provide heterogeneous nucleation interfaces, modify local ion distribution and mobility, and influence crystal attachment and growth (Visscher and Stolz, 2005; Dupraz et al., 2009; Pedone et al., 2020).

Importantly, this microbial effect was not proportional to biomass but followed a distinct triphasic trajectory comprising pre-optimal, optimal, and post-optimal regimes (Figures 6A, B). This nonlinear response suggests that increasing biomass progressively modifies both the availability of potential nucleation sites and the conditions controlling subsequent crystal growth. According to classical nucleation theory, mineral precipitation depends not only on supersaturation but also on the energetic barrier to nucleation and the availability of favorable nucleation interfaces (Kashchiev, 2000; Mullin, 2001; Reznik et al., 2009). Heterogeneous nucleation on pre-existing surfaces can lower the energetic barrier relative to homogeneous nucleation, providing a theoretical basis for considering microbial cell surfaces and EPS as potential nucleation interfaces.

The pre-optimal phase (Figures 6A, B), associated with low biomass and extended incubation, exhibited a low recovered salt precipitate mass, slow operational crystallization rates, and small, poorly individualized subcubic crystals. These crystals were also relatively heterogeneous and closely aggregated, indicating limited crystal development (Figures 7A–A2, D–D2, 8: SB-0.5 and BB-0.5). This reflects nucleation site limitation due to insufficient biomass and constrained EPS production, resulting in partial utilization of supersaturation and slow crystal growth, consistent with classical nucleation theory where activation energy is insufficiently compensated by active nucleation centers (Kashchiev, 2000; Mullin, 2001; Reznik et al., 2009). More specifically, the limited abundance of cell- and EPS-associated surfaces at low biomass may reduce the availability of favorable interfaces for heterogeneous nucleation, thereby constraining the number of nuclei that can develop despite the presence of supersaturation. Interestingly, relatively high specific yields observed in this phase suggest a dilution effect, where production per biomass remains substantial despite low total halite formation. These relatively high specific yields should nevertheless be interpreted cautiously, as normalization against a very low biomass input may generate apparently high values without necessarily indicating greater absolute crystallization efficiency.

The optimal regime (Figures 6A, B) represents a dynamic equilibrium between nucleation density and crystal growth, resulting in maximal halite yield and precipitation rates. Crystallization rates peaked at 0.552 g day^−1^ in SB approximately 0.297–0.299 g day^−1^ in BB, with the highest observed BB value reaching 0.299 ± 0.028 g day^−1^ (Figures 6C, D; Supplementary Table S3), coinciding with the formation of larger, well-defined crystals and well-faceted halite crystals, with greater individualization and population homogeneity. These crystals display subhedral to euhedral morphologies (Figures 7B–B2, E–E2, 8: SB-25 and BB-75), indicative of sustained, organized growth under low kinetic constraints. EPS play a critical role as nucleation scaffolds, stabilizing microgradients and facilitating mineral–microbe interface organization (Arp et al., 2001; Dupraz et al., 2009; Pace et al., 2016; Visscher et al., 2022). At intermediate biomass, greater abundance of microbial surfaces and EPS-associated interfaces may therefore provide a favorable balance between heterogeneous nucleation and continued ion transport toward growing crystal surfaces. EPS-mediated microscale gradients in ion concentration and saturation state may further promote nucleation and support sustained crystal growth (Braissant et al., 2009; Decho, 2010). This behavior aligns with microbial biomineralization models in diverse evaporitic environments, where biological activity optimizes supersaturation utilization and drives efficient crystal growth (Reid et al., 2000; Bosak and Newman, 2003; Braissant et al., 2007). Conversely, the post-optimal phase (Figures 6A, B), observed at high inoculum levels, showed that further increases in biomass did not enhance halite-dominated precipitate mass but were instead associated with stabilization or a decline in precipitation, poorer crystal development, greater morphological heterogeneity, and increased crystal aggregation, characterized by variable crystal sizes, less regular cubic to subcubic morphologies, and less sharply defined edges (Figures 7C–C2, F–F2, 8: SB-100 and BB-100). One possible explanation is that an increased abundance of potential nucleation surfaces may favor the formation of numerous small crystals, which could compete for dissolved ions and potentially limit individual crystal growth, thereby contributing to the fine, disordered textures observed, consistent with saturation-dependent nucleation models (Mullin, 2001; Reznik et al., 2009). Thus, increasing biomass beyond the optimum may increase the number of simultaneous nucleation events without proportionally increasing the amount of dissolved material available for subsequent crystal growth. The available ions may consequently be distributed among a larger population of developing crystals, favoring finer and less organized textures. High microbial densities may further potentially impose diffusive constraints and locally modify chemical microenvironments, which could contribute to competition and ionic imbalance (Visscher and Stolz, 2005). Dense cellular and EPS-rich matrices may additionally potentially restrict ion diffusion toward growing crystal surfaces, such that the same biological matrix that may favors nucleation at intermediate biomass may become limiting for crystal enlargement at high biomass (Decho, 2010; Dupraz et al., 2009; Pace et al., 2016).

This trend is supported by Figure 6, which illustrates the biomass-dependent halite precipitation and crystallization kinetics across both surface- and bottom-brine enrichment cultures. The results identify optimal biomass conditions associated with higher crystal yields and well-developed morphologies, in contrast to non-optimal conditions characterized by reduced precipitation efficiency.

Thus, the nonlinear response to biomass likely reflects a progressive shift from insufficient nucleation at low biomass, to an optimal balance between nucleation and crystal growth at intermediate biomass, and finally to competing nucleation, ion competition, and potential diffusional limitations at high biomass (Figure 6).

Extracellular polymeric substances (EPS) may provide a potential link between microbial biomass and the observed crystallization patterns. By providing reactive organic surfaces that interact with dissolved ions, EPS could potentially favor heterogeneous nucleation and support crystal growth at intermediate biomass levels. Conversely, excessive biomass and a potentially greater abundance of EPS-rich material may be associated with conditions favoring the formation of numerous smaller crystals rather than the development of individual crystals. EPS may therefore act as a potential interface between microbial biomass and local geochemical conditions. However, because EPS production and composition were not directly measured, this mechanism remains a plausible hypothesis rather than a demonstrated causal process. Similarly, the potential influence of irradiation on EPS-mediated processes cannot be confirmed without direct measurements.

#### 5.3.2 Surface-bottom brine differentiation in halite crystallization

Our findings showed that crystallization followed a triphasic trajectory in both SB and BB, comprising pre-optimal, optimal, and post-optimal regimes; however, the characteristics and intensity of these phases differed between the two inoculum types. SB-derived cultures generally exhibited higher halite-dominated precipitate mass and faster crystallization across most of the tested biomass range (Supplementary Table S3). The maximum crystallization rate in SB was approximately twice as high as that observed in BB (Figures 6A, B). Furthermore, SB reached its maximum crystallization performance at an intermediate biomass level, whereas BB required substantially higher biomass to reach its highest observed rate. These differences indicate that inoculum origin affected not only the amount of salt precipitated but also the kinetics of crystallization and the biomass level required for optimal performance.

Although both inocula originated from the same pond (TS5), SB and BB represented distinct vertical microhabitats corresponding to the surface and bottom layers near the sediment. Their different crystallization responses therefore suggest that microbial abundance alone cannot explain the observed behavior and that the ecological origin of the inoculum may influence the efficiency of mineral formation.

This pattern mirrors the vertical physicochemical gradients that can develop within hypersaline brines, particularly with respect to oxygen availability, light exposure, temperature, redox conditions, and associated microbial activity (Oren, 2002; Braissant et al., 2007, 2009; Elloumi et al., 2009; Bourhane et al., 2023; Pace et al., 2016). Similar stratification effects have been documented in hypersaline lakes, where aerobic phototrophs dominate surface layers and support enhanced mineralization compared to anoxic sediments (Oren, 2001; Gunde-Cimerman et al., 2018).

Community composition further explains the functional divergence observed. SBs were enriched with phototrophic and pigmented halophiles, including *Dunaliella salina* and carotenoid-rich haloarchaea, which may influence local physicochemical conditions through their metabolic activity and adaptation to intense solar irradiation (Oren, 2001; Bourhane et al., 2023; Gammoudi et al., 2022). Phototrophic activity can modify local pH, oxygen availability, and dissolved inorganic carbon, potentially generating microscale chemical gradients that influence microbial interactions and mineral precipitation. These organisms also produce osmolytes and metabolites such as glycerol, which serve as substrates for heterotrophic communities and reinforce trophic interactions and biofilm structuring (Kulp et al., 2007; Decho, 2010; Weng et al., 2025). In contrast, BBs experience anoxic conditions, lower energy availability, and dominance of anaerobic metabolisms, which may influence microbial activity, extracellular matrix production, and the availability of biologically favorable interfaces for heterogeneous nucleation. These ecological contrasts are consistent with observations in the Sfax salterns, where community structure varies markedly between the water column and sediments, the latter hosting higher microbial diversity but exhibiting distinct biomineralization activity (Baati et al., 2008, 2010; Trigui et al., 2011; Boujelben et al., 2012).

The vertical separation between SB and BB may therefore expose the resident communities to different combinations of light, oxygen availability, temperature, salinity, nutrient supply, organic matter, and redox conditions. Such environmental differences can influence microbial physiology and community composition and, consequently, the functional properties of the inocula used in crystallization experiments. The distinct crystallization responses observed between SB and BB may thus reflect the combined effects of vertical physicochemical gradients and differences in community functionality rather than biomass alone.

Light and oxygen gradients are particularly relevant to this vertical differentiation. Surface-associated communities are directly exposed to solar radiation and may contain phototrophic and pigment-rich microorganisms. The Sfax solar salterns harbor diverse extremely halophilic microorganisms, including *Haloquadratum, Halorubrum, Halobacterium, Haloferax*, and *Haloarcula*, as well as the red-pigmented bacterium *Salinibacter ruber* (Baati et al., 2008; Trigui et al., 2011; Boujelben et al., 2012). Phototrophic microorganisms such as *Dunaliella salina, Chlamydomonas rubrifilum*, and halophilic cyanobacteria have also been reported under highly saline conditions. Haloarchaea commonly accumulate C_50_ carotenoids dominated by bacterioruberin, whereas *S. ruber* produces salinixanthin. Halobacterium *salinarum* isolated from the Sfax salterns has been reported to produce bacterioruberin and related carotenoids under salinities comparable to those investigated here (Baati et al., 2022; Ben Hamad Bouhamed et al., 2024).

Such pigment-rich and phototrophic communities could influence crystallization indirectly through photosynthetic modification of local pH, oxygen availability, and dissolved inorganic carbon. CO_2_ uptake during illumination can increase pH, whereas respiration during dark periods may partially reverse these gradients, generating microscale chemical heterogeneity that could influence microbial interactions and mineral precipitation (Dupraz et al., 2004; Ludwig et al., 2005; Maza-Márquez et al., 2022; Weng et al., 2025). Pigment-associated absorption of solar radiation may also contribute to photoprotection and localized heating, potentially affecting microbial activity and, under intense irradiation, evaporation and NaCl supersaturation (Oren, 2001; Bourhane et al., 2023; Gammoudi et al., 2022).

Oxygen availability may provide an additional distinction between the two source habitats. Oxygen solubility decreases with increasing salinity, while restricted gas exchange and microbial respiration can promote more reducing conditions in deeper brines and sediment-associated layers (Oren, 2002; Braissant et al., 2007, 2009; Elloumi et al., 2009; Bourhane et al., 2023; Pace et al., 2016). Similar vertical stratification has been described in hypersaline environments, where oxygenated surface waters coexist with deeper layers characterized by different redox conditions and metabolic processes (Oren, 2001; Gunde-Cimerman et al., 2018). Such gradients may influence microbial physiology and metabolic interactions and thereby alter the chemical microenvironment surrounding mineral formation.

The bottom-associated community is also more closely associated with the brine-sediment and mineral interface. Sfax saltern sediments harbor diverse archaeal and bacterial assemblages, including *Haloarcula, Halococcus, Natronomonas, Halomonas, Marinobacter, Alcanivorax, Pseudomonas*, and *Salinivibrio*, together with uncultured microbial lineages (Baati et al., 2010). Molecular analyses identified 64 archaeal and 32 bacterial OTUs in Sfax saltern sediments, highlighting the substantial phylogenetic diversity of this compartment (Baati et al., 2010). Such communities may differ functionally from surface-associated populations and may therefore contribute to the distinct crystallization response of BB.

Metabolic interactions may further contribute to these differences, as osmolytes and metabolites released by halophilic phototrophs can support heterotrophic microorganisms and promote extracellular matrix formation (Kulp et al., 2007; Decho, 2010; Weng et al., 2025). Collectively, these observations suggest that the contrasting SB–BB responses may reflect the combined effects of vertical physicochemical gradients and differences in community functionality rather than biomass alone.

#### 5.3.3 Integrated model, limitations, and future perspectives

Taken together, the results support a coupled conceptual model of halite crystallization in which evaporation establishes the thermodynamic conditions for mineral precipitation, whereas microbial communities may influence the subsequent crystal development and morphological organization of halite. Within this framework, microbial effects on nucleation and crystal growth are proposed as possible mechanisms underlying the observed patterns, rather than as directly demonstrated processes. The comparison with the abiotic control demonstrates that evaporation alone is sufficient to drive precipitation, whereas the much higher rates observed in microbial cultures show that microbial communities can substantially accelerate this process. The triphasic biomass response further suggests that this microbial contribution is not simply proportional to biomass and may reflect an optimum balance between processes that potentially favor crystal initiation and processes that may limit subsequent crystal growth. The involvement of enhanced nucleation and growth limitation remains hypothetical because these processes were not directly measured in the present study. The contrasting SB and BB responses demonstrate that this balance is also influenced by inoculum origin and, consequently, by the ecological characteristics of the source community. Thus, the present findings do not support a strict distinction between “abiotic” and “biotic” halite formation but rather indicate a hierarchical interaction in which evaporation provides the primary geochemical driving force, while microbial communities modulate the kinetics and organization of mineral formation (Oren, 2002; Dupraz et al., 2009; Warren, 2016; Pedone et al., 2020).

Several limitations should nevertheless be acknowledged. The enrichment cultures represent selected fractions of the natural microbial communities, and the contribution of individual taxa or functional groups could not be resolved. Although the strong representation of *Halorubrum* in M3 cultures is consistent with its adaptation to the applied hypersaline conditions, it does not demonstrate a direct role in enhanced crystallization. Similarly, the higher performance of SB-derived cultures cannot be attributed to a specific phototrophic, archaeal, or bacterial group because community composition and functional activity were not characterized at the resolution required to establish causality. EPS abundance and composition were not directly measured, while oxygen, light intensity, temperature, and microscale redox conditions were not monitored during crystallization. Consequently, mechanisms involving EPS-mediated nucleation, pigment-associated effects, phototrophic activity, and metabolic modification of microscale chemical gradients remain plausible but unverified and should therefore be considered possible explanations for the observed differences in crystallization behavior. Direct measurements of EPS production and composition, microscale chemical gradients, pigment activity, and nucleation and growth processes would be required to test these hypotheses.

Future studies should therefore extend the present laboratory approach to *in situ* experiments in the Sfax solar salterns, integrating measurements of key physicochemical parameters including salinity, temperature, pH, dissolved oxygen, redox potential, light intensity, evaporation, and major ions with microbial community profiling, metabolic activity, pigment and EPS characterization, and direct monitoring of crystal nucleation, growth, mineralogy, and morphology. Such a multidisciplinary field-based approach would help establish causal links between environmental conditions, microbial community functions, and crystallization dynamics and clarify the specific mechanisms through which microbial communities contribute to natural halite formation.

## 6 Conclusion

This study demonstrates that halite crystallization in the Sfax solar salterns results from the interplay between brine geochemistry, environmental conditions, and microbial communities. While physicochemical conditions drive NaCl precipitation, microbial biomass may modulate crystal nucleation, growth kinetics, and morphology. Three archaeal strains affiliated with *Halorubrum*, isolated from surface brines, were enriched under high-salinity conditions (280 g L^−1^), suggesting that these archaea, together with other microorganisms and environmental factors, may contribute to shaping the crystallization environment. Surface and bottom-brine communities exhibited distinct effects on halite precipitation, with a reproducible triphasic response (pre-optimal, optimal, and post-optimal). Optimal biomass promoted well-defined euhedral crystals, whereas low or excessive biomass favored disordered microcrystalline aggregates, indicating that microbial effects depend on both biomass abundance and inoculum characteristics. Overall, these findings provide new insights into the coupling between halophilic microbial ecology and evaporite mineralization. Further validation under natural *in situ* conditions is required to determine the extent to which these microbial effects operate in natural evaporitic systems.

## Data Availability

The data analyzed in this study are publicly available in the NCBI GenBank database under accession numbers PZ224748, PZ224749, and PZ224750.
