# Supplementary material for: Halite crystallization at Sfax solar salterns, Tunisia, driven by brine geochemistry and biogeochemical interactions
Source: Front Microbiol. 2026 Oct 13;17:1843317. doi: 10.3389/fmicb.2026.1843317 (PMC13712413; doi:10.3389/fmicb.2026.1843317)
Supplement: Supplementary file 1 [file Supplementary_file_1.docx]

**Table S1.** Physicochemical characteristics of brine samples collected along the salinity gradient of the Sfax-Thyna solar saltern (Tunisia). Major ion concentrations are expressed in g L⁻¹

| Site | Basin  type | Facies | CO₃²⁻  (g l^-1^) | HCO₃⁻  (g l^-1^) | Ca  (g l^-1^) | SO₄²⁻  (g l^-1^) | Na  (g l^-1^) | Cl  (g l^-1^) | Mg  (g l^-1^) | K  (g l^-1^) | Density | pH | T (°C) | TSS  (g l^-1^) | Salinity  (g l^-1^) | Mean salinity  ± SD (g l-1) | NaCl equivalent (g L⁻¹) | | Mean NaCl equivalent ± SD (g L⁻¹) |
| --- | --- | --- | --- | --- | --- | --- | --- | --- | --- | --- | --- | --- | --- | --- | --- | --- | --- | --- | --- |
| SW | Sea | DF | 0.002 | 0.015 | 0.468 | 2.962 | 12.000 | 3.701 | 1.472 | 0.463 | 1.026 | 8.64 | 25 | 0.0 | 21.083 | 28.19 ± 10.21 | 15.701 | 21.42 ± 8.2 | |
| Wi |  |  | 0.002 | 0.015 | 0.426 | 3.044 | 12.333 | 3.810 | 1.396 | 0.463 | 1.021 | 8.57 | 26 | 0.0 | 21.489 |  | 16.143 |  |  |
| A2 | PEP |  | 0.002 | 0.012 | 0.511 | 3.332 | 13.000 | 4.485 | 1.430 | 0.513 | 1.023 | 8.20 | 25 | 0.0 | 23.285 |  | 17.485 |  |  |
| A5 |  |  | 0.002 | 0.015 | 0.553 | 3.456 | 13.333 | 4.606 | 1.447 | 0.513 | 1.024 | 8.46 | 26 | 0.0 | 23.925 |  | 17.939 |  |  |
| A11 |  |  | 0.006 | 0.024 | 0.617 | 4.484 | 16.667 | 7.500 | 1.919 | 0.657 | 1.032 | 9.12 | 25 | 0.0 | 31.874 |  | 24.167 |  |  |
| A17 |  |  | 0.006 | 0.024 | 0.787 | 6.254 | 23.333 | 13.756 | 2.463 | 0.850 | 1.045 | 8.76 | 25 | 0.0 | 47.473 |  | 37.089 |  |  |
| C21 | EP | OCF | 0.006 | 0.024 | 0.915 | 6.254 | 23.333 | 14.577 | 2.395 | 0.900 | 1.046 | 8.80 | 25 | 0.0 | 48.404 | 84.55 ± 34.25 | 37.910 | 63.91 ± 27.97 | |
| C22 |  |  | 0.003 | 0.027 | 0.447 | 8.599 | 32.500 | 27.643 | 17.982 | 1.217 | 1.067 | 8.25 | 26 | 0.0 | 88.418 |  | 60.143 |  |  |
| C23 |  |  | 0.002 | 0.027 | 1.511 | 12.507 | 42.500 | 51.178 | 7.394 | 1.717 | 1.096 | 8.02 | 27 | 0.1 | 116.836 |  | 93.678 |  |  |
| M21 | IP | GCF | 0.003 | 0.031 | 1.426 | 13.083 | 44.167 | 57.859 | 8.670 | 1.867 | 1.103 | 8.17 | 26 | 0.0 | 127.106 | 179.35 ± 49.07 | 102.026 | 151.60 ± 46.05 | |
| M22 |  |  | 0.003 | 0.037 | 1.213 | 14.358 | 51.667 | 107.183 | 9.478 | 2.267 | 1.123 | 8.28 | 27 | 0.1 | 186.206 |  | 158.850 |  |  |
| M23 |  |  | 0.003 | 0.052 | 1.064 | 15.922 | 63.333 | 130.603 | 10.817 | 2.933 | 1.160 | 8.16 | 24 | 0.1 | 224.727 |  | 193.936 |  |  |
| B21 | 50-H | GF | 0.003 | 0.055 | 0.830 | 18.308 | 64.150 | 148.870 | 11.051 | 3.300 | 1.165 | 7.97 | 24 | 0.1 | 256.567 | 249.65 ± 13.97 | 213.020 | 213.42 ± 11.70 | |
| B22 |  |  | 0.002 | 0.049 | 0.638 | 17.650 | 68.325 | 133.592 | 10.052 | 3.267 | 1.176 | 7.89 | 24 | 0.1 | 233.575 |  | 201.917 |  |  |
| B23 |  |  | 0.001 | 0.052 | 0.681 | 17.691 | 68.325 | 156.984 | 8.819 | 3.267 | 1.177 | 7.91 | 25 | 0.1 | 258.820 |  | 225.309 |  |  |
| PM2 | MB | GHF | 0.000 | 0.052 | 0.553 | 18.102 | 81.650 | 202.936 | 11.328 | 3.533 | 1.184 | 7.89 | 24 | 0.0 | 318.154 | 338.65 ± 19.05 | 284.586 | 303.06 ± 17.16 | |
| PM4 |  |  | 0.000 | 0.055 | 0.489 | 19.583 | 85.000 | 221.268 | 12.582 | 3.700 | 1.195 | 7.89 | 24 | 0.2 | 342.677 |  | 306.268 |  |  |
| PM6 |  |  | 0.000 | 0.064 | 0.468 | 19.871 | 86.650 | 231.669 | 12.603 | 3.800 | 1.201 | 7.91 | 28 | 0.1 | 355.125 |  | 318.319 |  |  |
| TS11 | ST | HF | 0.000 | 0.177 | 0.213 | 50.851 | 70.000 | 484.514 | 34.287 | 9.833 | 1.245 | 7.62 | 28 | 0.5 | 649.875 | 516.412 ± 110.361 | 554.514 | 439.81 ± 115.86 | |
| TS21 |  |  | 0.000 | 0.098 | 0.340 | 29.869 | 85.000 | 352.958 | 19.874 | 5.900 | 1.228 | 7.64 | 24 | 0.3 | 494.039 |  | 437.958 |  |  |
| TS3 |  |  | 0.000 | 0.092 | 0.319 | 27.071 | 90.000 | 339.951 | 16.919 | 5.367 | 1.219 | 7.83 | 26 | 0.1 | 479.719 |  | 429.951 |  |  |
| TS38 |  |  | 0.000 | 0.153 | 0.234 | 41.923 | 75.000 | 439.912 | 28.314 | 8.333 | 1.235 | 7.90 | 26 | 1.5 | 593.869 |  | 514.912 |  |  |
| TS5 |  |  | 0.000 | 0.220 | 0.234 | 54.307 | 70.000 | 191.690 | 37.242 | 10.867 | 1.247 | 7.65 | 27 | 0.9 | 364.560 |  | 261.690 |  |  |
| R3 | RB | HPF | 0.000 | 0.156 | 0.213 | 39.661 | 83.300 | 469.085 | 28.335 | 8.000 | 1.231 | 7.87 | 25 | 0.1 | 628.750 | 567.766 ± 220.374 | 552.385 | 459.74 ± 239.81 | |
| S2 |  |  | 0.000 | 0.229 | 0.213 | 54.718 | 70.000 | 525.627 | 36.073 | 10.667 | 1.249 | 7.92 | 25 | 0.1 | 697.527 |  | 595.627 |  |  |
| S4 |  |  | 0.000 | 0.226 | 0.340 | 60.190 | 65.000 | 532.285 | 40.112 | 11.633 | 1.254 | 7.55 | 25 | 0.6 | 709.786 |  | 597.285 |  |  |
| S8 |  |  | 0.000 | 0.378 | 0.043 | 47.272 | 14.150 | 79.507 | 85.648 | 8.000 | 1.278 | 6.67 | 30 | 2.7 | 235.0 |  | 93.657 |  |  |
| L1 | LB |  | 0.000 | 0.458 | 0.043 | 32.954 | 4.150 | 5.146 | 102.910 | 1.833 | 1.315 | 6.18 | 34 | 0.3 | 147.494 | 147.494 ± 2.5 | 9.296 | 9.296 ± 1.72 | |

TSS: total suspended solids; PEP: Pre-evaporation Ponds; EP: External Partition; IP: Internal Partition; 50-H: The 50-Hectare Area; MB: Main Basins; ST: Salt Tables; RB: Reserve Basins and LB: and “L” Basin, DF: Detrital facies; OCF: Organo-carbonate facies; GCF: Gypsum-carbonate facies; GF: Gypsum facies; GHF: Gypsum-Halite facies; HF: Halite facies; and HPF: Halite-potash facies

**Table S2.** Microbial diversity along the salinity gradient (35–450 g L⁻¹) in the Sfax solar salterns, Tunisia.

| Domain | Microorganism / taxon | Ecological  group | Sampling  site | Sample type | Salinity range (g L⁻¹) | Reference |
| --- | --- | --- | --- | --- | --- | --- |
| Archaea | *Natronomonas* sp. | Extremely halophilic archaeon | TS18 | Brine | 150–300 | Boujelben et al. (2012) |
|  | *Haloterrigena thermotolerans* | Extremely halophilic archaeon | Sfax salterns | Brine | 200–390 | Boujelben et al. (2014) |
|  | *Haloferax* sp. | Extremely halophilic archaeon | Sfax salterns | Brine | ~200 | Boujelben et al. (2014) |
|  | *Haloquadratum walsbyi* | Extremely halophilic archaeon | TS38 / S5 / TS18 | Brine | 250–360 | Baati et al. (2008); Boujelben et al. (2012) |
|  | *Haloquadratum* sp. | Extremely halophilic archaeon | TS | Brine | ~390 | Trigui et al. (2011) |
|  | *Halorubrum tibetense* | Extremely halophilic archaeon | TS38 / S5 | Brine | 250–310 | Baati et al. (2008) |
|  | *Halorubrum chaoviator* | Extremely halophilic archaeon | TS18 | Brine / sediment | ~390 | Ghanmi et al. (2016, 2020) |
|  | *Halorubrum ezzemoulense* | Extremely halophilic archaeon | TS18 | Brine | 380–390 | Frikha Dammak et al. (2016) |
|  | *Halorubrum* sp. | Extremely halophilic archaeon | TS38 / TS / TS18 | Brine / sediment | 250–390 | Baati et al. (2008); Trigui et al. (2011); Ghanmi et al. (2016, 2020) |
|  | *Halobacterium salinarum* | Extremely halophilic archaeon | TS18 / TS7 | Brine / sediment | ~284–390 | Ghanmi et al. (2016, 2020); Abbes et al. (2013) / Bouhamed et al. (2024) |
|  | *Halobacterium halobium* | Extremely halophilic archaeon | TS7 | Brine | ~284 | Abbes et al. (2013) |
|  | *Halonotius pteroides* | Extremely halophilic archaeon | TS18 / R2 / S6 | Brine | 290–360 | Boujelben et al. (2012) |
|  | *Halogeometricum* sp. | Extremely halophilic archaeon | TS18 / R2 / S6 | Brine | 290–360 | Boujelben et al. (2012) |
|  | *Haloarcula* sp. | Extremely halophilic archaeon | TS38 | Sediment | 250–300 | Baati et al. (2010) |
|  | *Halococcus* sp. | Extremely halophilic archaeon | TS38 | Sediment | 250–300 | Baati et al. (2010) |
|  | *Natronomonas pharaonis* | Extremely halophilic archaeon | TS38 | Sediment | 250–300 | Baati et al. (2010) |
|  | Candidate division MSBL-1 | Archaeal lineage | TS38 | Sediment | 250–300 | Baati et al. (2010) |
|  | MSP clones | Archaeal lineage / uncultured archaea | TS38 | Sediment | 250–300 | Baati et al. (2010) |
| Bacteria | *Salinivibrio costicola* | Moderately halophilic bacterium | TS38 | Sediment | 50–150 | Baati et al. (2010) |
|  | *Vibrio* sp. | Halophilic bacterium | TS38 | Sediment | 50–150 | Baati et al. (2010) |
|  | *Alcanivorax* sp. | Halophilic bacterium | TS38 | Sediment | 50–150 | Baati et al. (2010) |
|  | *Marinobacter* sp. | Halophilic bacterium | TS38 | Sediment | 50–150 | Baati et al. (2010) |
|  | *Halomonas venusta* | Moderately halophilic bacterium | TS38 | Sediment | 50–250 | Baati et al. (2010) |
|  | *Halomonas* sp. | Halophilic bacterium | TS38 | Sediment | 50–250 | Baati et al. (2010) |
|  | *Pseudomonas stutzeri* | Halophilic bacterium | TS38 | Sediment | 150–300 | Baati et al. (2010) |
|  | *Pseudomonas halophila* | Halophilic bacterium | TS38 | Sediment | 150–300 | Baati et al. (2010) |
|  | *Phyllobacterium* sp. | Heterotrophic bacterium | TS | Brine | 150–200 | Trigui et al. (2011) |
|  | *Salinibacter ruber* | Extremely halophilic bacterium | TS38 / S5 / TS18 | Brine/sediment | 250–360 | Baati et al. (2008, 2010); Boujelben et al. (2012) |
|  | *Salinibacter* sp. | Extremely halophilic bacterium | TS | Brine | ~390 | Trigui et al. (2011) |
|  | *Pseudaminobacter* sp. | Halophilic/heterotrophic bacterium | TS38 | Sediment | 250–300 | Baati et al. (2010) |
|  | *Halorhodospira* sp. | Halophilic phototrophic bacterium | TS38 | Sediment | 250–300 | Baati et al. (2010) |
|  | *Alkalilimnicola* sp. | Halophilic bacterium | TS18 / R2 / S6 | Brine | 290–360 | Boujelben et al. (2012) |
|  | Bacteroidetes (uncultured) | Halophilic heterotrophic bacteria | TS18 / R2 / S6 | Brine | 290–360 | Boujelben et al. (2012) |
|  | *Salicola* sp. | Extremely halophilic bacterium | TS18 | Brine/sediment | ~390 | Ghanmi et al. (2016, 2020) |
|  | *Lentibacillus halophilus* | Halophilic bacterium | TS18 | Brine/sediment | ~390 | Ghanmi et al. (2016, 2020) |
| Cyanobacteria | *Euhalothece* sp. | Halophilic cyanobacterium | TS38 / S5 | Brine | ~250–310 | Baati et al. (2008) |
|  | *Phormidium versicolor* | Halophilic cyanobacterium | High-level crystallizers | Brine | 200–450 | Gammoudi et al. (2022) |
|  | *Aphanothece* sp. | Halophilic cyanobacterium | High-level crystallizers | Brine | 200–450 | Gammoudi et al. (2022) |
|  | *Spirulina subsalsa* | Halophilic cyanobacterium | High-level crystallizers | Brine | 200–450 | Gammoudi et al. (2022) |
|  | *Navicula* sp. | Diatom | Sfax salterns | Brine | 35–80 | Abid et al. (2008); Ayadi et al. (2002, 2004) |
| Eukarya | *Nitzschia longissima* | Diatom | Sfax salterns | Brine | 35–80 | Abid et al. (2008); Ayadi et al. (2002, 2004) |
|  | *Gymnodinium* sp. | Dinoflagellate | Sfax salterns | Brine | 35–200 | Abid et al. (2008); Ayadi et al. (2002, 2004) |
|  | *Prorocentrum* sp. | Dinoflagellate | Sfax salterns | Brine | 35–200 | Abid et al. (2008); Ayadi et al. (2002, 2004) |
|  | *Halamphora* sp. SB1 | Diatom | Sfax salterns | Brine | 45–80 | Abid et al. (2008) |
|  | *Ochromonas* sp. | Chrysophyte | Sfax salterns | Brine | 70–110 | Abid et al. (2008) |
|  | *Dunaliella salina* | Chlorophyte / halophilic microalga | High-level crystallizers | Brine | 200–450 | Gammoudi et al. (2022) |
|  | *Chlamydomonas rubrifilum* | Chlorophyte/microalga | High-level crystallizers | Brine | 200–450 | Gammoudi et al. (2022) |
|  | *Artemia salina* | Crustacean/zooplankton | Sfax salterns | Brine | 150–350 | Toumi et al. (2005); Guermazi et al. (2008); Ladhar et al. (2014) |
|  | *Fabrea salina* | Ciliate/bacterivore | Sfax salterns | Brine | 150–350 | Elloumi et al. (2006, 2009) |
| Fungi | *Eurotium rubrum* | Halophilic/halotolerant fungus | TS18 | Associated hypersaline microbial environment | ~390 | Ghanmi et al. (2016, 2020) |
|  | *Penicillium corylophilum* | Halophilic/halotolerant fungus | TS18 | Associated hypersaline microbial environment | ~390 | Ghanmi et al. (2016, 2020) |
|  | Fungi, taxa not specified | Fungal component | High-level crystallizers | Brine | 200–450 | Gammoudi et al. (2022) |
| Viruses | Halophilic viruses / haloviruses | Viral component; microbial lysis | TS / TS18 | Brine | ~390 | Trigui et al. (2011); Ghanmi et al. (2016, 2020) |
|  | Viral activity/lysis | Ecological process; microbial population control | TS / TS18 | Brine | ~390 | Trigui et al. (2011); Ghanmi et al. (2016, 2020) |

**Table S3.** Inoculum characteristics and salt precipitation and crystallization parameters in SB- and BB-derived cultures.

| Samples | Inoculum | | | Salt  (mean ± SD, g) | Duration  (mean, d) | Crystallization rate (mean ± SD, g d⁻¹) |
| --- | --- | --- | --- | --- | --- | --- |
|  | Inoculum  (% v/v) | Wet biomass (mg) | Wet biomass concentration (mg mL⁻¹) |  |  |  |
| SB-0.5 | 0.5 | 2.22 ± 0.09 | 0.444 ± 0.018 | 2.515 ± 0.064 | 18 | 0.140 ± 0.015 |
| SB-1 | 1 | 3.37 ± 0.14 | 0.674 ± 0.028 | 2.760 ± 0.071 | 18 | 0.154 ± 0.008 |
| SB-5 | 5 | 21.50 ± 0.88 | 4.300 ± 0.176 | 3.325 ± 0.049 | 16 | 0.209 ± 0.015 |
| SB-10 | 10 | 38.40 ± 1.57 | 7.680 ± 0.314 | 3.350 ± 0.113 | 18 | 0.186 ± 0.006 |
| SB-25 | **25** | **109.00 ± 4.47** | **21.800 ± 0.894** | **5.550 ± 0.127** | **10** | **0.555 ± 0.013** |
| SB-50 | 50 | 219.00 ± 8.98 | 43.800 ± 1.796 | 3.810 ± 0.028 | 12 | 0.318 ± 0.002 |
| SB-75 | 75 | 328.90 ± 13.48 | 65.780 ± 2.696 | 4.100 ± 0.156 | 11 | 0.375 ± 0.034 |
| SB-100 | 100 | 440.00 ± 18.04 | 88.000 ± 3.608 | 3.195 ± 0.021 | 17 | 0.188 ± 0.001 |
| BB-0.5 | 0.5 | 2.14 ± 0.09 | 0.428 ± 0.018 | 2.120 ± 0.071 | 18 | 0.118 ± 0.004 |
| BB-1 | 1 | 3.28 ± 0.13 | 0.656 ± 0.026 | 3.095 ± 0.007 | 17 | 0.183 ± 0.015 |
| BB-5 | 5 | 21.40 ± 0.88 | 4.280 ± 0.176 | 2.205 ± 0.035 | 18 | 0.123 ± 0.012 |
| BB-10 | 10 | 36.80 ± 1.51 | 7.360 ± 0.302 | 1.475 ± 0.050 | 18 | 0.082 ± 0.009 |
| BB-25 | 25 | 100.10 ± 4.10 | 20.020 ± 0.820 | 2.690 ± 0.028 | 15 | 0.179 ± 0.002 |
| BB-50 | 50 | 209.00 ± 8.57 | 41.800 ± 1.714 | 2.510 ± 0.099 | 16 | 0.157 ± 0.006 |
| BB-75 | **75** | **321.00 ± 13.16** | **64.200 ± 2.632** | **3.570 ± 0.085** | **12** | **0.299 ± 0.028** |
| BB-100 | 100 | 428.00 ± 17.55 | 85.600 ± 3.510 | 2.855 ± 0.035 | 15 | 0.191 ± 0.016 |
| T− | **0** | **0** | **0** | **1.640 ± 0.014** | **45** | **0.036 ± 0.001** |

**Figure S1.** Piper trilinear diagram (Piper and Garrett, 1953) illustrating the hydrochemical composition and relative distribution of major cations and anions in the different water bodies and total brine samples.

**Figure S2**. Z-score-normalized heatmap illustrating hydrochemical variability among brine samples from the M22, B22, and TS5 ponds.

**Figure S3.** (A) Six culture media inoculated with surface brine (SB) and bottom brine (BB) samples collected from basins M22, B22, and TS5, using three media formulations (M1, M2, and M3) with salinities of 150, 220, and 280 g L⁻¹, respectively. (B) Optical microscopy observations of the six culture conditions: (a) M1-SB, (b) M2-SB, (c) M3-SB, (d) M1-BB, (e) M2-BB, and (f) M3-BB (scale bar = 0.5 cm). (C) Two series of eight culture media (M3; 280 g L⁻¹) prepared from TS5 samples and independently inoculated with TS5-BB and TS5-SB at concentrations of 0.5%, 1%, 5%, 10%, 25%, 50%, 75%, and 100%. All cultures were incubated at 37 °C under continuous shaking (160 rpm).
